# Structure learning for gene regulatory networks

**DOI:** 10.1371/journal.pcbi.1011118

**Published:** 2023-05-18

**Authors:** Anthony Federico, Joseph Kern, Xaralabos Varelas, Stefano Monti

**Affiliations:** 1 Section of Computational Biomedicine, Boston University School of Medicine, Boston, Massachusetts, United States of America; 2 Bioinformatics Program, Boston University, Boston, Massachusetts, United States of America; 3 Department of Biochemistry, Boston University School of Medicine, Boston, Massachusetts, United States of America; bioinformatics, GERMANY

## Abstract

Inference of biological network structures is often performed on high-dimensional data, yet is hindered by the limited sample size of high throughput “omics” data typically available. To overcome this challenge, often referred to as the “small *n*, large *p* problem,” we exploit known organizing principles of biological networks that are sparse, modular, and likely share a large portion of their underlying architecture. We present SHINE*—***S**tructure Learning for **Hi**erarchical **Ne**tworks—a framework for defining data-driven structural constraints and incorporating a shared learning paradigm for efficiently learning multiple Markov networks from high-dimensional data at large *p/n* ratios not previously feasible. We evaluated SHINE on Pan-Cancer data comprising 23 tumor types, and found that learned tumor-specific networks exhibit expected graph properties of real biological networks, recapture previously validated interactions, and recapitulate findings in literature. Application of SHINE to the analysis of subtype-specific breast cancer networks identified key genes and biological processes for tumor maintenance and survival as well as potential therapeutic targets for modulating known breast cancer disease genes.

## Author summary

Network biology is a systems-based approach to modeling complex biological mechanisms. Modeling systems of interacting molecular entities—such as genes, proteins, and metabolites, among others–provide a semantically rich representation for understanding biological diseases and other phenomena. Network reconstruction is often hampered by the small number of omics profiles available relative to the number of molecular entities, and by the need to reconstruct multiple networks representing distinct phenotypic groups. In this article, we present a novel and multi-pronged approach to overcoming these challenges by using biological organizing principle to limit the model parameters of structure learning and encourage shared learning of multiple networks. This approach limits the computational search space of possible network structures and takes advantage of a-priori structural information to increase the equivalent sample size, resulting in higher overall performance. While we focus on human gene regulatory networks, this method can be applied to and adapted for studying other biological systems and data types.

This is a *PLOS Computational Biology* Software paper.

## Introduction

Biological networks can model functional relationships at different cellular levels–genes, proteins, metabolites–and can be integrated to depict system-wide connectivity. Gene regulatory network (GRN) reconstruction aimed at inferring putative mechanistic interactions associated with disease phenotypes can support the identification of drivers of disease severity and treatment response [1–3]. Importantly, changes in network connectivity across experimental conditions or phenotypes may help pinpoint important context-specific regulators or mediators, and inform functional experiments aimed at elucidating mechanisms of action (MOAs), targetable vulnerabilities, and resistance to treatment [4–7].

GRNs represent the underlying structure that dictate functional properties of biological systems. The dependencies between genes provide insight into the regulatory mechanisms that drive biological phenomena. Markov networks—undirected graphical models satisfying the Markov property, i.e., capable of distinguishing between direct and indirect dependencies–support a semantically rich representation of GRNs. Multiple approaches have been proposed to model the distinction between direct and indirect dependencies, including Bayesian networks [8], information-theoretic approaches[9], deconvolution approaches[10], and feature selection approaches [11,12]. We present a novel inference framework that uses a Markov network representation based on a multivariate Gaussian model. Markov networks differ from Bayesian networks in that the latter are defined as *directed* acyclic graphs. Given the lack of a clear directionality of the probabilistic influence in high-dimensional observational data, adoption of a Markov network representation is deemed preferable, and less likely to lead to the erroneous interpretation of the edges’ directionality as the direction of causal effects. While Markov network-based methods have been previously proposed [11,13–20], they tend not to scale well, and often omics studies forgo Markov graphical modeling due to the unavailability of the large number of samples required for their inference in high-dimensional domains. Comparing networks across multiple phenotypic groups or time points further reduces already limited sample sizes, presenting an even greater challenge for Markov network inference from omics data.

**Fig 1:**
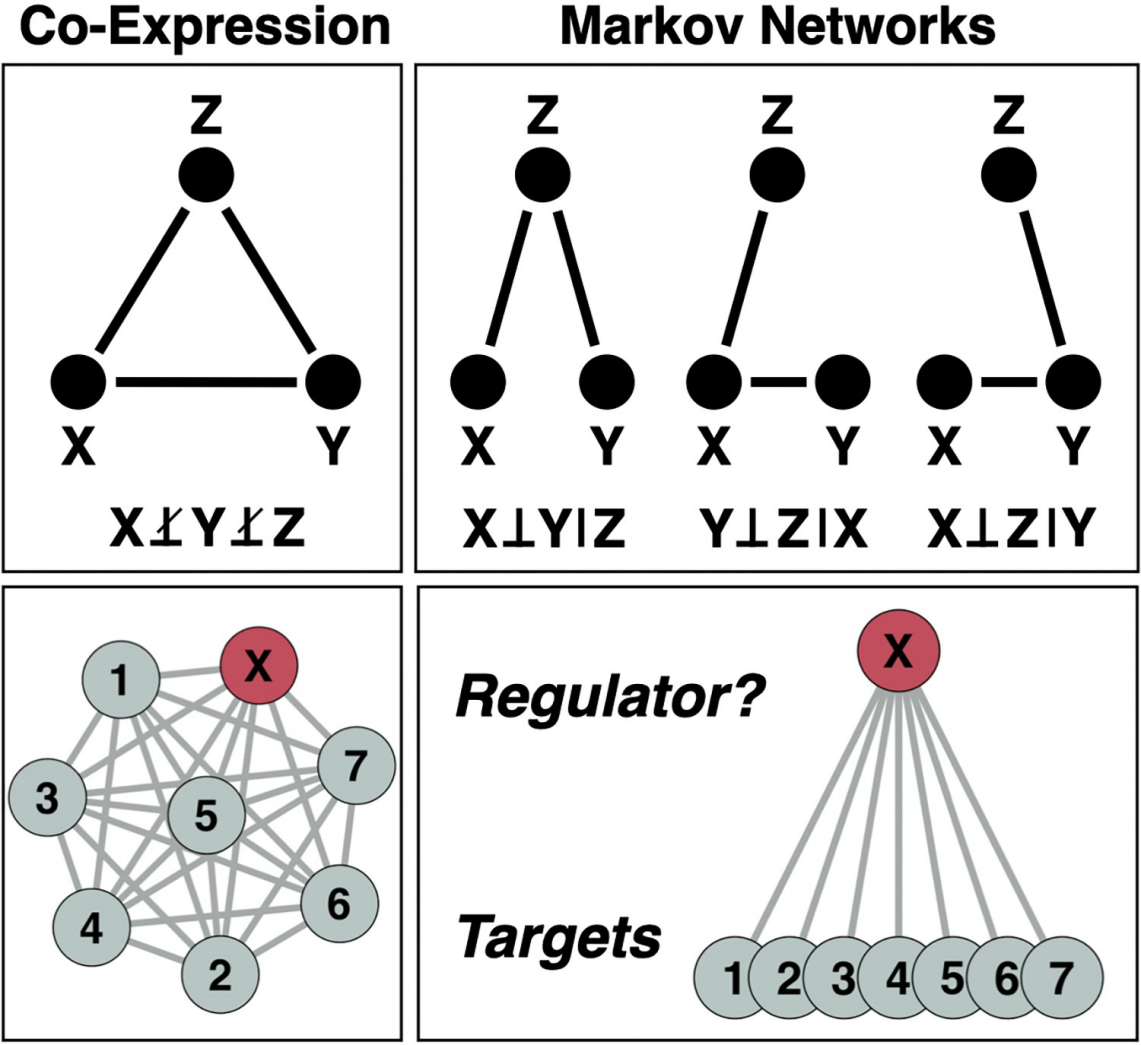
Co-Expression vs. Markov Networks. *Top*: The same co-expression structure may arise from multiple conditional independence structures. Notation: X⊥Y|Z means X independent of Y given Z. *Bottom*: A putative regulator is harder to identify in a co-expression network than a Markov network.

Inference of GRNs is an extremely active field of research, characterized by the frequent development of new methods and approaches, as well as collaborative initiatives to standardize their evaluation using a variety of data inputs, such as the DREAM challenges [21]. Here we focus on inferring GRNs from *observational data*, whereby the biological variation that exists across samples reflects the observed variation in a population rather than the results of experimental manipulations. In this context, gene connectivity is often inferred through pairwise correlation across genome-wide expression data. While a co-expression matrix is straightforward to compute, it fails to distinguish direct from indirect dependencies between genes, a distinction that a Markov network is best suited to model (Fig 1). To infer direct effects, one must consider the partial correlation of genes, that is, the correlation of two genes conditioned on all others. To that end, in linear settings the partial correlation or precision matrix Ω needs to be computed, which is intractable in high dimensional domains. Conventionally, the maximum-likelihood method, a frequentist approach to estimate model parameters that most likely explain the data, is used to estimate Ω. However, when *p > n–*i.e., when the number of variables *p* is larger than the sample size *n*, as is the case for typical genome-wide omics data–the estimation of Ω becomes unstable and inaccurate. This is an instance of what is often referred to as the “small *n*, large *p* problem” [22,23].

Several regularization approaches have been developed to improve the estimation of Ω, such as the graphical lasso and its extensions [11,14,15], yet these improvements are not sufficient to enable the inference of large networks from small biological datasets. An alternative method for learning Markov networks is a Bayesian approach to search for likely graphs that encode the data [16,24]. Bayesian structure learning also enables the incorporation of prior beliefs in the graphical search. However, even this approach is still insufficient to achieve accurate inference when *n*≪*p*.

In this report, we develop and evaluate SHINE (**S**tructure Learning for **Hi**erarchical **Ne**tworks), a multi-pronged approach to Markov network reconstruction that combines Bayesian inference with constraint learning–to incorporate well-established modular properties of biological networks–and with shared learning–to pool information across networks representing related domains (e.g., tumor subtypes). In doing so, we limit the search complexity and increase the equivalent sample size for graphical modeling of high dimensional biological data, and we are thus able to learn networks at *p/n* ratios not matched by any other methods we tested.

Due to an exponentially expanding solution space in structure learning–with 2^*p*(*p*-1)/2^ possible graphs to choose from–finding appropriate constraints to limit the number of graphs to consider drastically improves inference, especially for high dimensional models. More specifically, as entities in the cell–such as genes, proteins, metabolites–form co-regulated modules expected to share regulatory programs [25], we use this modular structure to form a high-level representation of an integrated regulatory space by estimating the modules’ interdependencies. We can thus reduce the complexity of the graphical search space by first identifying module-based constraints, and by then ruling out unlikely inter-module gene interactions. Additionally, when learning multiple networks representing specializations of a common domain, it is important to consider evidence that their topology is partially conserved–even between evolutionarily distinct species–particularly for genes involved in core biological processes [26]. Thus, a shared learning approach where data is shared between related phenotypes can be used to address underpowered analysis of high dimensional biological datasets with a small number of samples [15,27–29].

We evaluate SHINE on simulated network structures representative of known biological properties and compare its performance to eight existing network detection methods. We also apply SHINE to the reconstruction of 23 tumor-specific networks from The Cancer Genome Atlas (TCGA) data, and present an in-depth case study where SHINE is incorporated in an analysis workflow focused on breast cancer and on the identification of candidate therapeutic vulnerabilities.

## Results

### SHINE Algorithm overview

The SHINE algorithm takes a multi-pronged approach to learning biological Markov networks, whereby multiple related networks are learned in a hierarchical procedure. First, structure learning constraints are applied based on co-expression *module detection*, to identify genes unlikely to be interacting and thus to reduce the complexity of the graphical search space. Second, a *network hierarchy* is defined based on the relationships between groups of samples representing distinct phenotypes from which networks are to be learned. The network hierarchy, the detected modules, and the omics dataset are the inputs to the learning procedure, which is outlined in S1 Fig. Using a top down approach, child networks take advantage of a-priori structural information from previously learned parent networks in the hierarchy (S4 Fig). Structure constraints are detected and applied at the root level of the hierarchy and used in a divide and conquer (DAQ) fashion, whereby subgraphs (from the feature sets of extended modules) are learned independently and then merged to create a final global network structure. When learning a child network from a parent network in the DAQ-context, the posterior distribution of edges of each parent subgraph are used as a prior in learning the child subgraphs, which are then reconstructed into a final child network structure. Each of the networks is estimated based on a birth-death Markov chain Monte Carlo algorithm for the inference of undirected GGM using marginal pseudo-likelihood maximization [24].

### Constrained learning limits the structure search space

A well-accepted approach for reducing the complexity of gene-based models is to reduce the dimensionality of the data to co-expression modules. Given a *p x n* gene expression matrix *X*, by taking advantage of the zero-order correlations between expression profiles *x*_*i*_ and *x*_*j*_ across *n* samples, genes can be organized into *m* co-expression modules of sizes *p*_*1*_,…, *p*_*m*_, such that $\sum_{i}p_{i}=p$. Markov network inference can then be *mainly* constrained to within each of the lower dimensional *m* modules. This is based on the principle that complex biological systems have a modular structure and gene regulatory networks carry out biological functions through units of coordinated expression[30–32]. Accounting for network modularity before Markov network inference allows one to first consider genes likely to be interacting based on co-expression similarity, which can be computed with few samples. To this end, many methods have emerged to detect network modules based on gene co-expression[33]. The simplest approach is to detect mutually exclusive—or *isolated*—modules and to then constrain the graphical search to intra-modular interactions. A range of approaches can then be defined to *extend* these modules, based on varying criteria of stringency in constraining the inter-modular relationships to be modeled.

### Isolated modules

Using methods popularized in Weighted Correlation Network Analysis (WGCNA) [34] and following consistent notation [35], genes are clustered by their co-expression similarity *s*_*ij*_−measured by the absolute value of the biweight midcorrelation [36] coefficient: *s*_*i*,*j*_ = | *bicor*(*x*_*i*_, *x*_*j*_) | as well as a soft thresholding value *ß* which pushes spurious correlations to zero, resulting in a symmetric *p x p* weighted adjacency matrix *a*_*ij*_ = *s*_*ij*_^*ß*^. Co-expression modules are detected using hierarchical clustering of a topological overlap dissimilarity transformation *d*_*i*,*j*_ of *a*_*ij*_ resulting in *Q* modules. These modules represent interacting functional units that serve as a reasonable first order approximation of the larger biological network organization.

**Fig 2:**
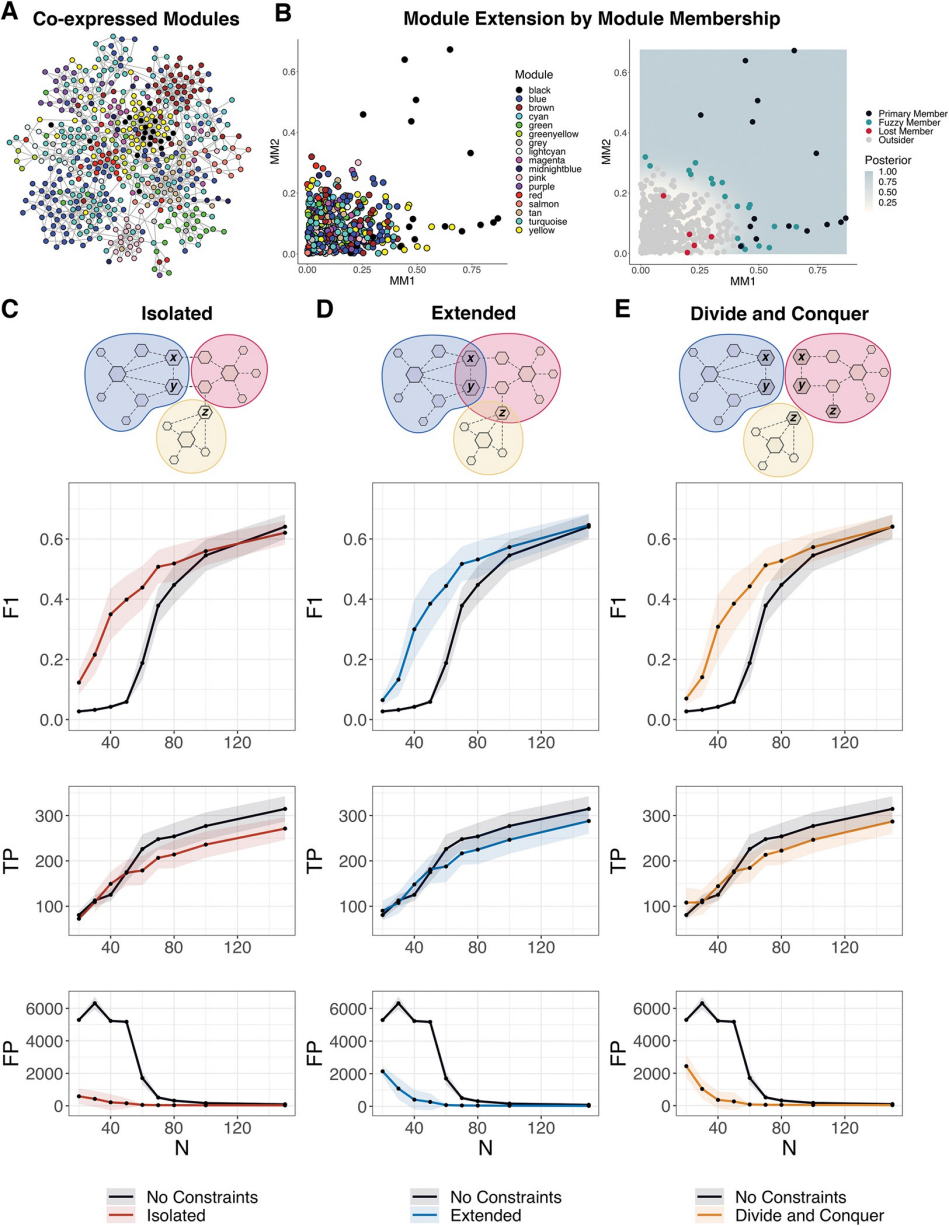
Module Constraints and Simulations. A) Detection of co-expression modules from simulated expression data where nodes are colored by their primary module. B) Nodes scored by their first (MM1) and second (MM2) membership in the black module (*left*). Nodes are assigned a membership probability *M*_*p*_ (posterior) to the black module based on MM1/MM2. Black module membership is extended to non-member nodes with *M*_*p*_ > 0.75 (Fuzzy Members) (*right*). C-E) Performance of structure learning using constraint-based methods on a single network (*p* = 300) across an increasing sample range from 20 to 150 evaluated by the mean and standard deviation (*iterations* = 25) of F1 score (F1), true positives (TP), and false positives (FP). Isolated (C), Extended (D), and Divide and Conquer (E) approaches were compared to a baseline performance where no constraints were used. Note the inclusion of genes *X*, *Y*, and *Z* in multiple modules in the schematic of the Divide and Conquer approach; edge *X–Y* will be included in the final combined network since it appears in both DAQ modules in which *X* and *Y* are members.

Here, we use them as a structural constraint when estimating the full graph, where the graphical search only considers the interaction of genes *i* and *j* if they belong to the same module (Fig 2A). However, ignoring inter-module interactions imposes a strict constraint and yields a disconnected global structure, which is contrary to our understanding that biological processes are interconnected [37]. Therefore, we consider inter-modular interactions by finding genes co-expressed across two or more modules. In doing so, we prioritize the inference of intra-module interactions and strongly correlated inter-module interactions.

### Extended modules

We utilize a previously established concept in module detection called module membership (*MM*) to allow modules to contain overlapping sets of genes [34]. Genes are assigned a membership score across all modules, where the membership of gene *i* in module *q* is the correlation of *i* with the module eigengene *E*^*(q)*^, which is the first principal component of the expression profiles of genes within *q*, thus *MM = | bicor(x*_*i*,_ *E*^*(q)*^*) |*. *MM* helps identify genes associated with multiple modules. Here we consider the *MM* of genes derived from the first (*MM1*) and second principal component (*MM2*), which together often account for a majority of the variation of genes within a given module (Fig 2B).

For each module, we consider non-member genes (outsiders) to become fuzzy members by comparing their first and second membership scores to primary member genes. This approach is formalized as a classification problem where we perform quadratic discriminant analysis to classify genes (with some membership probability, *M*_*p*_) (Fig 2B). In practice, control over the constraint levels is desirable. Thus, the probability of membership is a threshold that can be adjusted. After classification, modules are extended from their original members to the inclusion of fuzzy members. In this case, the graphical search only considers the interaction of genes *i* and *j* if they share one or more modules. Rather than constraining the entire graph, we employ a divide and conquer (DAQ) strategy for extended modules where we learn a graph for each module independently in parallel and then merge these subgraphs into a final network. Conceptually with this modular approach, we are prioritizing local dependencies, by only accepting inferred edges if they are valid under all local conditions or subgraphs where they are tested.

### Divide and conquer network inference

When building a global network structure from multiple subgraphs corresponding to extended modules based on the DAQ approach (Fig 2E), an edge between two nodes that are members of more than one extended module is included in the final network structure only if it appears in each of the constructed subgraphs (S1 Fig, lines 12–23). More precisely, an edge *X–Y* appears in a subgraph for module *M* if nodes *X* and *Y* have a non-zero partial correlation within that module, which means that *X* and *Y* are *not* independently conditioned on all the other genes in module *M*, formally, *¬X⊥Y| M*. If two nodes *X* and *Y* are members of extended modules *M*_*1*_, *M*_*2*_, *…*, *M*_*k*_, then the edge *X–Y* will be included in the combined network if and only if the edge *X–Y* appears in subgraphs for modules *M*_*1*_ through *M*_*k*_. This heuristic implies that if *¬X⊥Y|M*_*i*_, *i = 1*,..,*k*, then ¬X⊥Y|∪_*i*_ M_*i*_, which is clearly not true in general. However, our simulation studies show that this heuristic does not lead to an inflation of false positive edges, a point to which we now turn.

### Simulation studies

We conducted simulation studies to compare methods for constraint-based structure learning on a single network with 300 nodes using the Lancichinetti–Fortunato–Radicchi (LFR) benchmark [38]. Simulated graph structures were used to simulate multivariate Gaussian data for an increasing number of samples ranging from 20 to 150, with the maximum sample size (*n* = 150) still significantly smaller than the data dimensions (*p* = 300). Overall, the constraint-methods performed better (by F1) than without constraints (Default) (Fig 2C–2E). The constrained-based methods managed to significantly reduce the number of false positives (FP), that is, incorrectly estimated edges, particularly with smaller sample sizes, which is advantageous when we are primarily interested in detecting high confidence interactions to generate hypotheses that can be validated in an experimental setting. Additionally, these methods maintained true positive (TP) levels, i.e., correctly estimated edges, similar to those achieved with an unconstrained search, thus resulting in overall improved performance by F1. While constraints based on isolated modules had the fewest FP, the resulting disconnected global graph structure makes downstream network analysis challenging. Thus, we prefer constraints based on extended modules. The efficiency and scalability of constraint-based structure learning with extended modules was further improved by using the DAQ strategy without a loss of overall performance.

### Shared learning increases the equivalent sample size

When multiple networks are needed to model specializations of a common domain (e.g., different sub-types of breast cancer), encouraging shared learning of structural features is beneficial for each network individually. To implement this approach, we organize networks into a hierarchy, building a rooted tree of related networks. Leaf networks represent individual sample groups while networks higher in the hierarchy represent sample group supersets. Networks are inferred iteratively from the root down to the leaves, where at each level, the estimated posterior edges of the parent network are used to determine the prior distribution of edges in estimating the child network. The result is a hierarchy where internal and leaf networks represent shared and distinct network structures, respectively.

**Fig 3:**
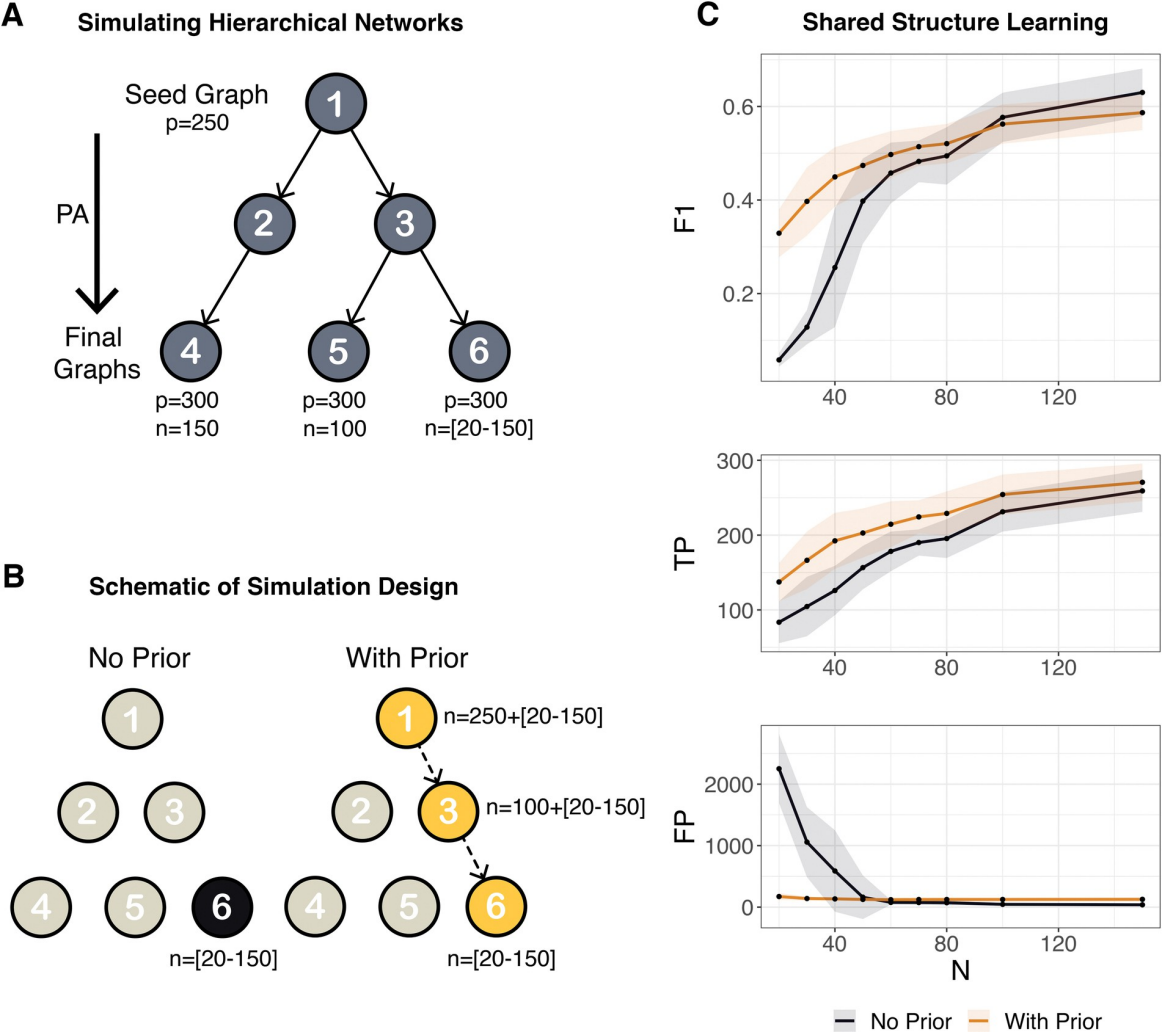
Simulation Studies for Shared Learning on Hierarchical Networks. A) A schematic of generating a network hierarchy by simulating independently diverging graph structures via preferential attachment (PA) with a known edge similarity, arriving at final graph structures used to simulate data for sample groups 4, 5, and 6. B) A schematic of the simulation design whereby shared learning of network 6 (using samples from group 6) incorporates prior information from network 3 (pooled samples from groups 5–6) which incorporates prior information of network 1 (pooled samples from groups 4–6). C) Performance of structure learning using the DAQ constraint-based method with and without prior information on network 6 across an increasing group 6 sample range from 20 to 150 evaluated by the mean and standard deviation (*iterations* = 25) of F1 score (F1), true positives (TP), and false positives (FP).

We simulated a hierarchy of similar networks to determine if shared learning further improves a constraint-based approach to network inference. To simulate network hierarchies, we employed an extension of the Barabási–Albert algorithm [39]—using the concepts of growth and preferential attachment—where multiple networks were simulated from a shared seed graph (modeled via LFR), resulting in a hierarchy of graphs with a known edge similarity (Fig 3A and 3B). With sample sizes at the lower extreme, we found the use of a prior network to be highly beneficial in addition to the incorporation of constraints (Fig 3C). Conversely, as *n* approached *p*, inference became tractable without constraints or prior information, shared learning limited the ability to detect new TP, and we saw diminishing returns in F1. This suggests that these methods are most applicable when sample size is small relative to the desired feature set, which is highly relevant in the context of biological network inference where *n*≪*p*. We refer to this use of constraint-based shared learning as SHINE.

### Comparing SHINE with Other methods

We compared SHINE to other available network inference methods in a network hierarchy context. In particular, we compared the accuracy of these methods in recovering the gold leaf network of the hierarchy in Fig 3B. Given our focus on the more difficult task of inferring Markov networks, capable of distinguishing between direct and indirect dependencies, we mainly focused our comparison on methods modeling this distinction. These include: Algorithm for the Reconstruction of Accurate Cellular Networks (ARACNe), which finds potential direct gene interactions by first estimating the mutual information between gene pairs, and by then applying the data processing inequality (DPI) to remove the weakest edge from every fully connected gene triplet; and Gene Network Inference with Ensemble of Trees (GENIE3) [12,40], which infers interactions by solving a regression problem for each gene. Both methods generate a ranked list of weighted interactions which can be used to construct a gene regulatory network by choosing an interaction threshold. We also compared SHINE to five Gaussian graphical model (GGM)-based methods specifically designed for high dimensional modeling: bivariate nodewise scaled Lasso (B_NW_SL) [17], de-sparsified graphical Lasso (D-S_GL) [18], de-sparsified nodewise scaled Lasso (D-S_NW_SL) [19], and GGM estimation with false discovery rate control with Lasso (GFC_L) and scaled Lasso (GFC_SL) [20]. Finally, as a baseline, we included a comparison to WGCNA, perhaps the most popular method for the inference of co-expression networks. Extensive evaluation and comparison of multiple co-expression network inference methods has been previously investigated [33].

Overall, SHINE had the highest performance of all the methods compared, with higher F1 scores across increasing sample sizes. SHINE had larger F1 improvement for small sample sizes (relative to the number of nodes) (Table 1). Importantly, the optimal thresholds of GENIE3, ARACNe, and WGNCA are not a-priori known. In contrast, SHINE’s adjacency matrices were constructed by a-priori setting a probabilistic threshold for edges (*p* = 0.9), with the results largely insensitive to the threshold choice (*p =* 0.85–0.95). This is preferred in real application settings, where the correct threshold is in general unknown.

**Table 1:** The Average F1-Score of Learned Networks with Increasing Sample Size. SHINE was compared to eight methods. Some methods required thresholding, each using various cut points for retaining the top percentage of predicted edges.

SHINE Comparison with Other Methods										
Method	Cut	n = 20	n = 30	n = 40	n = 50	n = 60	n = 70	n = 80	n = 100	n = 150
SHINE	-	0.329	0.397	0.449	0.474	0.497	0.514	0.521	0.562	0.587
GENIE3	0.1%	0.097	0.126	0.135	0.147	0.149	0.149	0.152	0.150	0.154
GENIE3	1.0%	0.222	0.304	0.355	0.391	0.413	0.436	0.444	0.469	0.505
GENIE3	2.5%	0.191	0.247	0.289	0.307	0.322	0.339	0.342	0.369	0.392
ARACNe	0.1%	0.121	0.137	0.147	0.153	0.152	0.156	0.157	0.156	0.156
ARACNe	1.0%	0.226	0.303	0.365	0.397	0.430	0.448	0.464	0.489	0.549
ARACNe	2.5%	0.027	0.090	0.108	0.136	0.148	0.168	0.195	0.165	0.198
WGCNA	0.1%	0.122	0.127	0.130	0.136	0.138	0.139	0.145	0.142	0.141
WGCNA	1.0%	0.259	0.314	0.336	0.376	0.387	0.398	0.409	0.414	0.437
WGCNA	2.5%	0.215	0.257	0.283	0.307	0.322	0.338	0.339	0.353	0.371
B_NW_SL	-	0.000	0.000	0.000	0.019	0.156	0.235	0.302	0.413	0.532
D-S_GL	-	0.000	0.024	0.108	0.200	0.267	0.320	0.376	0.422	0.524
D-S_NW_SL	-	0.044	0.132	0.217	0.277	0.324	0.365	0.395	0.473	0.553
GFC_L	-	0.145	0.228	0.305	0.357	0.399	0.427	0.452	0.517	0.582
GFC_SL	-	0.166	0.251	0.328	0.362	0.396	0.420	0.445	0.509	0.575

We next tested SHINE against the next-best performing method (GFC_SL) on the real TCGA Pan-Cancer data, learning networks for 1,157 cancer pathway genes across 23 tumor types with varying sample sizes (later described). We evaluated the performance of each method by their ability to recapture network edges that have been previously experimentally validated in human protein-protein interactions (PPI) databases. SHINE performed better on 20 of 23 networks with significantly improved performance on tumor types with few samples (S2 Fig). In line with the comparisons on simulated data, as *n* approaches *p*–in the case of BRCA–we find diminishing returns from SHINE’s strategy. Interestingly, the number of edges detected by SHINE is largely unaffected by sample size (*R*^*2*^ = 0.0618, *p* = 0.252) while the density of networks learned by GFC_SL is strongly correlated (*R*^*2*^ = 0.7703, *p* = 3.79e-08). On BRCA for example, GFC_SL detected 29,588 edges while SHINE detected 14,868 –which is closer to the expected number of edges identified by derived PPIs, 12,021. Densely connected networks may be more challenging to analyze, thus SHINE’s preference for high confidence interactions could be beneficial even when *n = p*.

### Evaluation on Pan-cancer data

We reconstructed Pan-Cancer networks from TCGA RNA-seq data, to evaluate SHINE and demonstrate its scalability. SHINE is particularly suitable to the study of cancer networks due to its assumption of partially conserved structure between network subtypes, a phenomenon documented in cancer [41]. Cancer types were organized into a four-level hierarchy, starting with all primary tumors (L0), then further stratified into organ system (L1), tumor type (L2), and tumor subtype (L3) (S1 Table) [42]. Due to our focus on cancer regulatory networks we learned networks from 1,157 genes involved in 14 major pathways encompassing hallmark capabilities of cancer (S2 Table) [43]. The rationale being that detection of changes in connectivity across such networks will likely represent differences in the outcome of cellular processes underlying tumor initiation and progression. We built extended modules on L0 and shared these constraints to learn L1 networks using the DAQ approach. L2 networks were learned using L1 as a prior based on the pre-defined hierarchy (S3 Fig).

**Fig 4:**
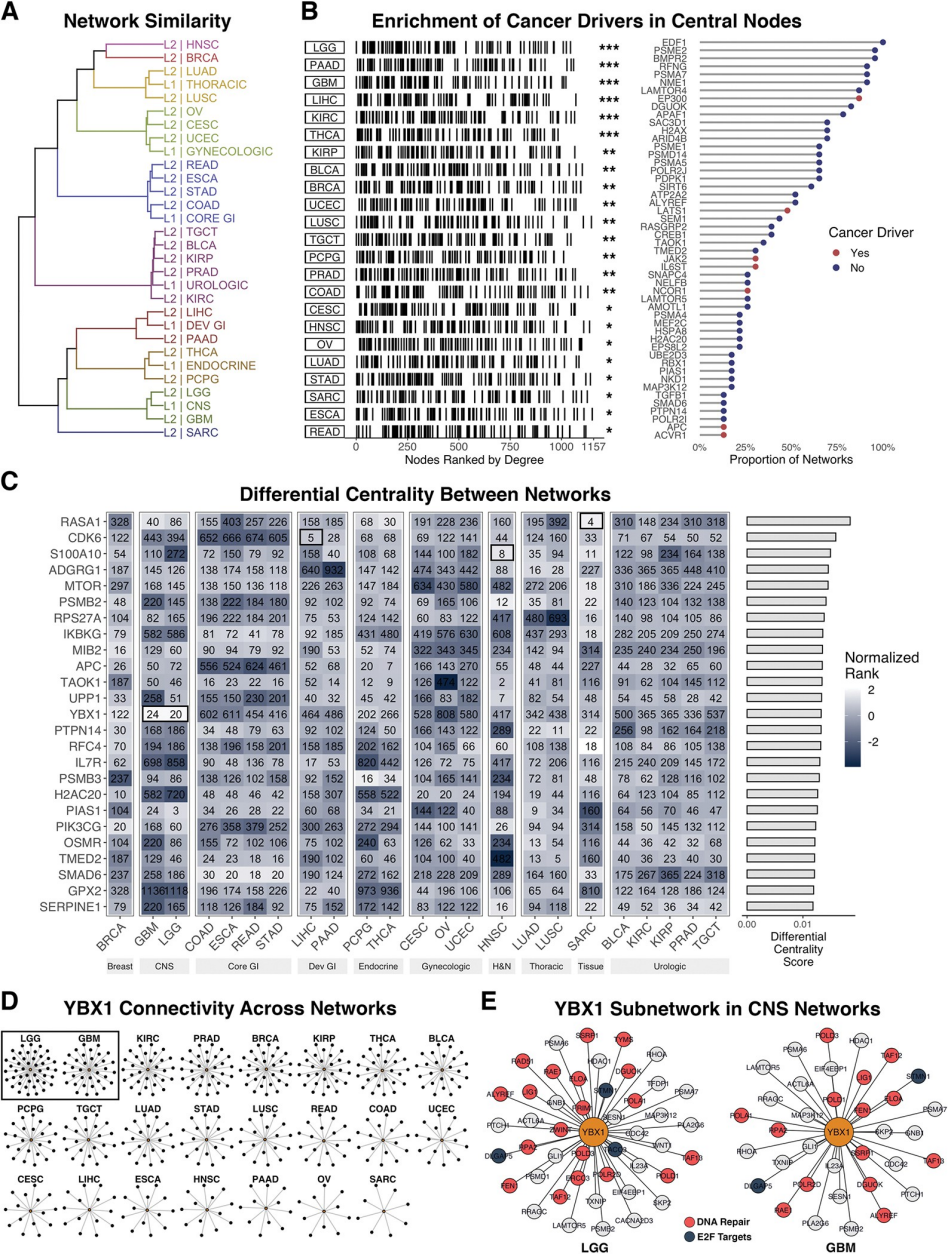
Pan-Cancer Networks. A) Network similarity clustered by Kendall rank correlation of degree centrality, where L1/L2 signify the network level in the pre-defined hierarchy. B) Enrichment of previously identified cancer driver genes in networks where nodes are ranked by degree (*left*) (*** = *p* < 0.005, ** = *p* < 0.01, * = *p* < 0.05). High-degree nodes (top 25) in at least one network. The top 50 most frequently present genes across networks are shown (*right*). C). Top 25 differentially central genes (by degree) between networks ranked by their differential centrality score. D) *YBX1* (*center-node*) and its first-degree connections in each network. E) *YBX1* subnetwork of first-degree connections in CNS networks (LGG and GBM).

We found networks largely clustered according to their prior network (e.g., L2 networks that shared an L1 network as a prior were more similar) (Fig 4A). We compared learned and randomly simulated networks (via *Barabási–Albert* [39] and *Erdős–Rényi* [44] models) to experimentally derived PPI [45]. Inferred interactions in each of the cancer networks were far more significantly enriched for PPIs than interactions in the random networks (BA and ER). Furthermore, learned networks shared similar graph properties with the PPI network, such as low density (sparsity)–here defined as having a number of edges much smaller than *E*_*max*_ (the maximum possible number of edges) [46]–and high global and local clustering coefficients (transitivity and clustering respectively), which are key properties of real biological networks (Table 2) [31].

**Table 2:** Common Graph-level and Node-level Measures Computed for Learned Pan-Cancer Networks. Inferred network edges were tested against a database of experimentally validated PPIs in human and compared with randomly simulated networks. Sample sizes for each network are denoted by *n* and all networks have a size of 1,157 nodes, including simulated and experimentally validated networks. TCGA study names for network abbreviations can be found in the S1 Appendix.

Network Properties and Comparison to Experimentally Validated PPIs									
Network	*n*	Edges	Density	Transitivity	Clustering	Assortivity	Overlap	P-Value	FDR
KIRC	538	14,868	0.022	0.125	0.170	0.115	638	4.8 x 10(-87)	1.2 x 10(-85)
KIRP	288	13,664	0.020	0.118	0.160	0.147	602	2.2 x 10(-86)	2.8 x 10(-85)
STAD	375	10,036	0.015	0.096	0.128	0.129	488	1.8 x 10(-83)	1.5 x 10(-82)
PRAD	498	14,163	0.021	0.120	0.167	0.121	604	2.2 x 10(-81)	1.4 x 10(-80)
BLCA	414	13,453	0.020	0.115	0.156	0.139	582	1.2 x 10(-80)	6.1 x 10(-80)
TGCT	150	13,119	0.020	0.113	0.155	0.132	567	2.1 x 10(-78)	8.7 x 10(-78)
READ	166	9,183	0.014	0.093	0.123	0.117	448	4.4 x 10(-77)	1.6 x 10(-76)
ESCA	161	9,048	0.014	0.092	0.125	0.143	443	1.1 x 10(-76)	3.4 x 10(-76)
COAD	478	10,790	0.016	0.101	0.137	0.113	491	8.6 x 10(-75)	2.4 x 10(-74)
THCA	502	10,977	0.016	0.097	0.128	0.103	480	6.5 x 10(-68)	1.6 x 10(-67)
LUAD	533	9,998	0.015	0.094	0.128	0.066	448	2.2 x 10(-66)	5.0 x 10(-66)
LIHC	371	7,449	0.011	0.077	0.104	0.118	370	1.0 x 10(-65)	2.1 x 10(-65)
LGG	511	10,557	0.016	0.100	0.129	0.115	456	4.8 x 10(-63)	9.2 x 10(-63)
PAAD	177	6,119	0.009	0.071	0.093	0.097	322	8.2 x 10(-63)	1.5 x 10(-62)
LUSC	502	9,489	0.014	0.092	0.125	0.078	423	4.0 x 10(-62)	6.6 x 10(-62)
UCEC	551	10,016	0.015	0.098	0.132	0.114	436	3.2 x 10(-61)	5.1 x 10(-61)
GBM	156	8,275	0.012	0.086	0.117	0.136	384	1.0 x 10(-60)	1.5 x 10(-60)
PCPG	178	8,607	0.013	0.08	0.108	0.111	393	2.3 x 10(-60)	3.2 x 10(-60)
OV	374	8,855	0.013	0.091	0.123	0.125	400	3.1 x 10(-60)	4.0 x 10(-60)
CESC	304	8,773	0.013	0.092	0.124	0.126	396	1.5 x 10(-59)	1.8 x 10(-59)
BRCA	1102	10,350	0.015	0.099	0.134	0.097	434	1.2 x 10(-56)	1.4 x 10(-56)
HNSC	500	6,578	0.010	0.078	0.109	0.085	309	3.9 x 10(-50)	4.4 x 10(-50)
SARC	259	3,639	0.005	0.061	0.081	0.049	172	7.2 x 10(-29)	7.8 x 10(-29)
BA^*1*^	NA	1,156	0.002	0.000	0.000	-0.116	30	3.2 x 10(-2)	3.3 x 10(-2)
ER^*1*^	NA	9,917	0.015	0.015	0.015	-0.002	179	5.0 x 10(-1)	5.0 x 10(-1)
PPI^*2*^	NA	12,021	0.018	0.196	0.277	-0.054	NA	NA	NA

^*1*^Simulated Networks

^*2*^Experimentally Validated

The degree distributions across networks were largely scale free, and highly connected nodes were enriched for known cancer drivers in all networks (*p =* 4.44e-16) (Fig 4B *left*). Notice that our use of a rank-based (Kolmogorov-Smirnov) procedure to test for enrichment protects us from over-estimating significance due to the biased set of genes initially selected for network construction. Highly connected nodes were conserved across a majority of the networks; many of which are implicated in the initiation, progression, and invasiveness of tumors. For example, *EDF1* was a top hub in every network and *PSME2* [47], *BMPR2* [48], *RFNG*, *PSMA7* [49], *NME1* [50], *LAMTOR4* [51], and *EP300* [52] were top hubs in at least 80% of networks. Interestingly, while cancer drivers tend to be more central across networks, the most connected hubs are not necessarily known cancer drivers (Fig 4B *right*).

To identify tumor type-specific differences, we scored nodes by their differential degree-centrality using a normalized rank score across networks. Many of the nodes found to be differentially central to a particular network(s) were previously implicated in their respective tumor type (Fig 4C). Using the top three as examples: *RASA1* was shown to be involved in the development of some sarcomas [53,54], CDK signaling was found to be a potential therapeutic target in hepatocellular carcinoma [55], and *S100A4* was shown to maintain cancer-initiating cells in head and neck cancers [56]. Interestingly, *YBX1*, a transcription factor involved in DNA repair, highly expressed in cancers, and known to promote cell growth and to inhibit differentiation in glial tumors is highly central in both CNS subtype networks (LGG and GBM) (Fig 4D) [57]. Furthermore, in these tumor types, *YBX1* subnetworks are enriched for DNA repair genes (*p* = 5.8e-07 and *p* = 4.3e-05; LGG and GBM respectively) and connected to multiple E2F targets (Fig 4E).

### Learning networks for breast cancer subtypes

**Fig 5:**
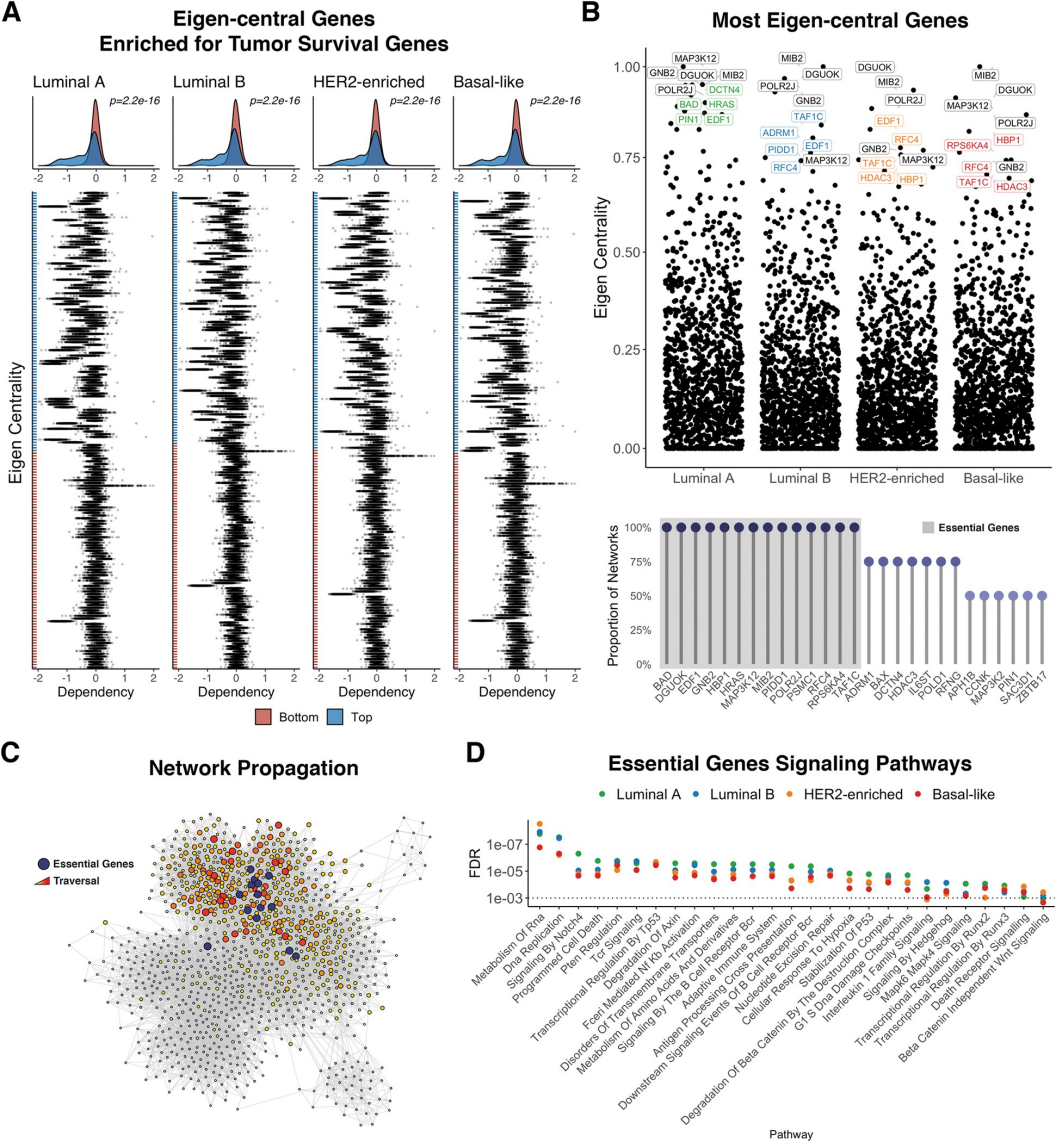
Breast Cancer Essential Genes. A) Genes within the top and bottom 100 eigen-centrality ranking across breast cancer networks scored by their genetic dependency across various cell lines via the Cancer Dependency Map Project (DepMap). A negative dependency value indicates higher essentiality to tumor survival. Genes not present in the DepMap are omitted. B) Top 10 eigen-central genes across networks (*top*). Top 25 eigen-central genes across multiple networks. Highlighted are 14 genes identified as highly central in all networks (*bottom*). C) A visualization of network propagation using the 14 eigen-central breast cancer genes as seed nodes where a red node indicates high traversal and close proximity to the seed genes. D) Biological pathway enrichment of highly traversed genes.

We applied SHINE to the analysis of TCGA Breast Cancer RNA-seq data to learn subtype-specific networks corresponding to the PAM50 molecular classification into Luminal A, Luminal B, HER2-enriched, and Basal-like (the Normal-like subtype was excluded because of too small a sample size, *n* = 40)[58]. To further boost the equivalent sample size, we used experimentally validated PPIs from breast cancer-related cell lines (e.g., MCF-7 and MDA-MB-231) as a structural prior or starting graph in the network hierarchy (S4 Fig) [59]. We found networks had similar community structure, and many of the top genes ranked by centrality measures were conserved across subtype networks (Fig 5B *top*). Interestingly, Luminal-B and HER2-enriched networks were most similar while Luminal-A was most dissimilar when comparing networks by centrality rankings.

We found eigen-central genes in the networks to be enriched for genes known to be essential for tumor survival identified by the Dependency Map Project (DepMap) (Fig 5A) [60]. An eigen-central node is not only highly connected, but also connected to other highly connected nodes, indicating high overall network influence [61]; thus, we expect highly eigen-central genes across inferred networks to play a central regulatory role in maintaining breast tumorigenesis and survival. Among these are 14 genes—*BAD*, *DGUOK*, *EDF1*, *GNB2*, *HBP1*, *HRAS*, *MAP3K12*, *MIB2*, *PIDD1*, *POLR2J*, *PSMC1*, *RFC4*, *RPS6KA4*, *TAF1C* - ranked within the top 25 by eigen-centrality in all four subtypes (Fig 5B *bottom*). Their highly conserved rank suggests they are likely to be central players driving and maintaining breast cancer. Interestingly, these central players localize in close proximity to each other in all network subtypes (Fig 5C). Network propagation seeded with these essential genes, and subsequent pathway enrichment analysis on highly traversed nodes suggests that these genes facilitate crosstalk between DNA maintenance/repair and immune-related pathways (Fig 5D).

**Fig 6:**
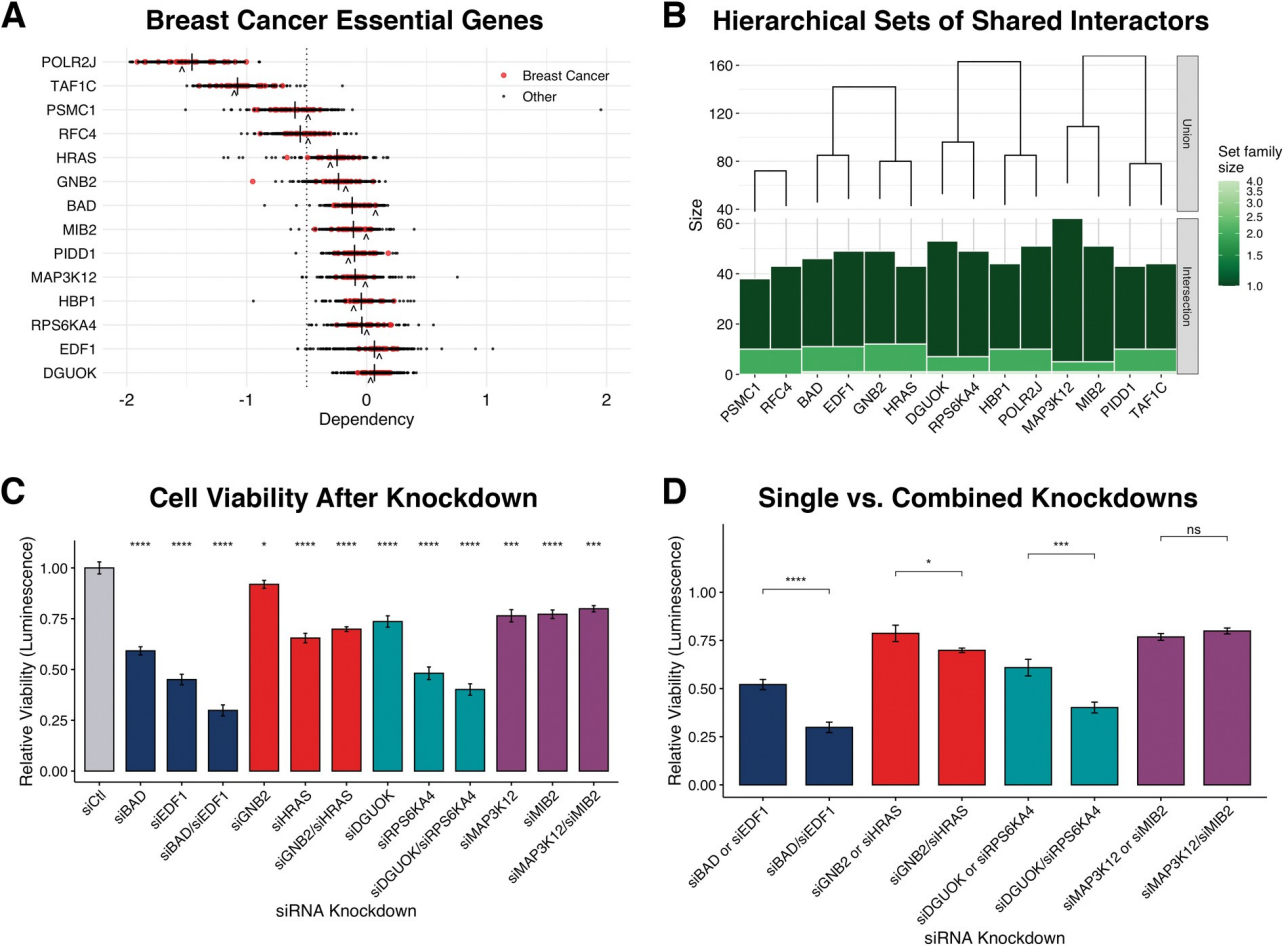
Validation of Breast Cancer Essential Genes. A) Breast cancer essential genes scored by their genetic dependency across breast cancer and other cell lines via the DepMap. The average dependency score across all cell lines (|) and MDA-MB-231 cells (^) are indicated. B) Gene pairs determined by intersecting sets of interactors. C) Cell viability relative to control (siCtl) after 4 days with single and paired gene knockdowns performed in MDA-MB-231 cells. Significance values were computed with a t-test for each knockdown compared to siCtl. D) Comparison of relative cell viability between single and combined knockdowns. Significance values were computed with a t-test for each single and combined knockdown pairs.

While eigen-central genes in the learned breast cancer networks were generally found to be essential for tumor survival by the DepMap, 10/14 highly eigen-central genes across all subtype networks were not, with an average dependency score > -0.5 across cell lines (Fig 6A). Because these genes share multiple interactions with other genes, we hypothesized redundant signaling pathways may allow for the loss of a single essential gene without cell death. We identified the most likely gene combinations by pairing genes that shared the most interactors (Fig 6B). We performed cell viability assays using the MDA-MB-231 cell line on pairs where both genes were not previously found to be essential to tumor survival in the DepMap. We confirmed gene silencing by siRNAs through RT-qPCR in MDA-MB-231 cells (S5 Fig). Knockdown of each gene alone resulted in significantly reduced cell viability (Fig 6C). Furthermore, combined knockdown of 3/4 pairs resulted in a significantly reduced cell viability compared to either gene alone. Pairs *BAD*/*EDF1* and *DGUOK*/*RPS6KA4* showed a dramatic combined effect decreasing cell viability by over 50% (Fig 6D).

**Fig 7:**
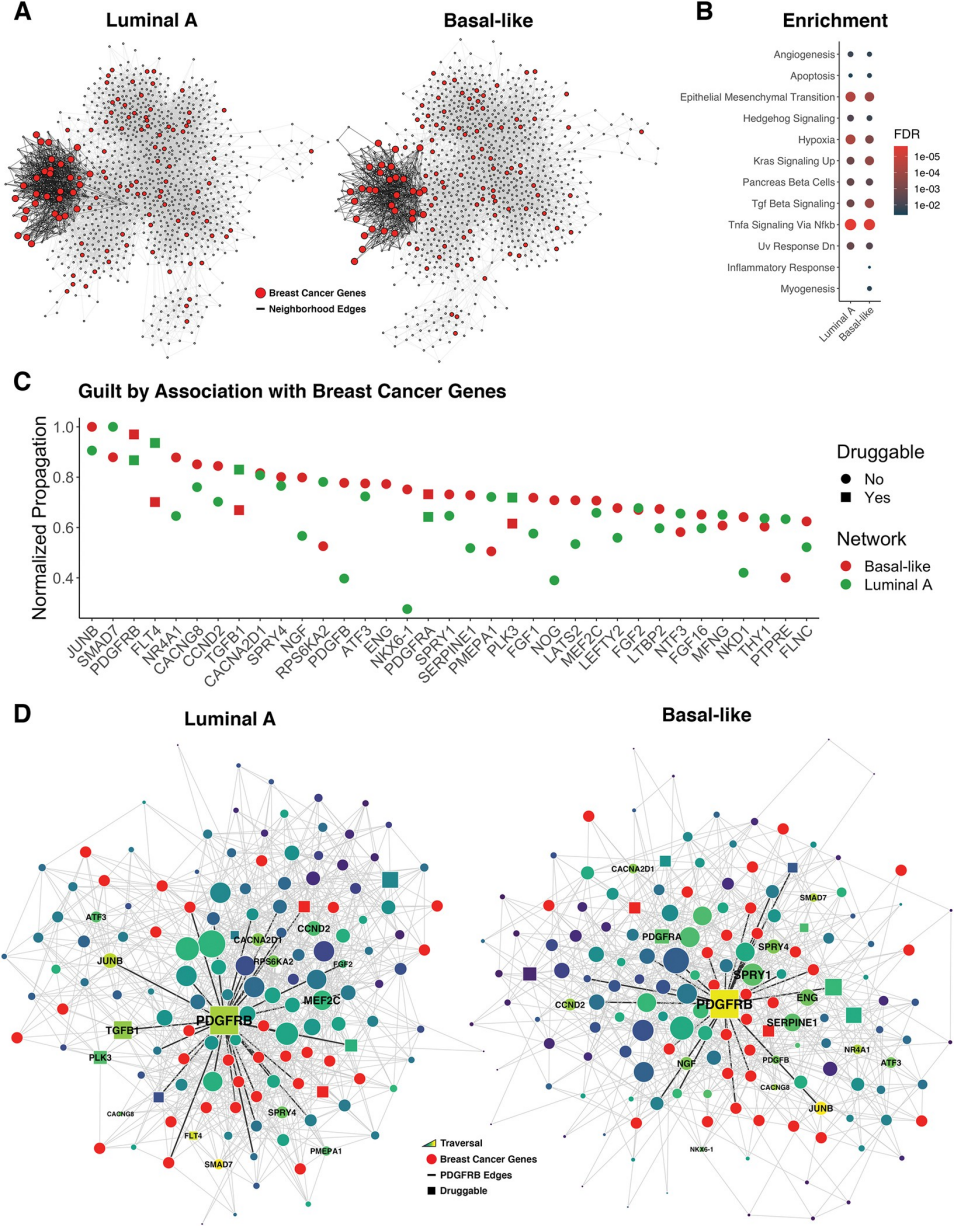
Breast Cancer Disease Neighborhood. A) A single community detected in breast cancer subtype networks enriched for known breast cancer disease genes. B) Pathway enrichment of the identified disease neighborhoods. C) Proximity of neighborhood genes to known breast cancer disease genes identified through network propagation. D) Subnetwork visualization of disease neighborhoods in Luminal A and Basal-like networks.

We next evaluated the use of the inferred networks to guide the identification of potential therapeutic targets. It has been postulated that drugs may be more effective through network-based actions targeting multiple disease genes [62]. Thus, we used the guilt-by-association principle to predict novel breast cancer genes by performing network propagation, seeded with the existing disease genes (Fig 7C and 7D) [63,64]. We found a community identified via Walktrap [65] community detection enriched for known breast cancer disease genes in Luminal A (*FDR* = 0.00035) and Basal-like (*FDR* = 0.0026) networks (Fig 7A) [66]. This disease neighborhood was present in both subtypes, containing the same set of 31 disease genes and enriched for immune-related signaling pathways (Fig 7B). Of note, not only is *PDGFRB* in close proximity to all disease genes in both networks, but it is also the most connected and eigen-central hub of the neighborhood. *PDGFRB* may be a prime candidate for disease modulation and is listed as a targetable gene (via Cediranib, Masitinib, Pazopanib) from the Genomics of Drug Sensitivity in Cancer [67]. Furthermore, *PDGF* signaling has been found to play an active role in breast tumor progression and *PDGFRB* has previously been theorized—along with its cognate ligand *PDGFB*—to be a potential therapeutic target for multiple human cancers, including breast [68]. Interestingly, *PDGFRB*/*PDGFB* are in closer proximity to disease genes in the Basal-like subnetwork further supporting evidence for its role in tumor aggressiveness [69].

## Discussion

In this report we present a novel multi-pronged approach called SHINE for learning biological Markov networks from limited sample sizes. We exploit known organizing principles of biological networks to limit the model parameters of structure learning and encourage shared learning of multiple networks to boost the equivalent sample size. This approach reduces the complexity of the search space, allows related networks to share data, and takes advantage of a-priori structural information, resulting in higher overall performance with fewer false positives. There is a cost of slightly more false negatives, however this tradeoff is advantageous in the context of biological network inference where *n*≪*p*, since we are primarily interested in predicting high confidence interactions for hypothesis generation. We apply SHINE to reconstruct tumor-specific networks from TCGA data as well as a focused analysis on breast cancer and find inferred networks exhibit expected graph properties of real biological networks, recapture previously validated interactions, and recapitulate findings in literature.

We found SHINE to be highly beneficial for learning regulatory networks, however this approach is only suitable when co-expression modules can be detected from the data. In simulation studies, we found that at very low samples sizes (*n* < 20 and *p* = 300), a failure to build appropriate constraints led to poor inference performance. While SHINE is largely data-driven and there are few parameters to optimize, it is useful to adjust the level of fuzziness in defining extended modules, which can be controlled via the threshold or decision boundary for secondary membership *M*_*p*_ (Fig 2B). *M*_*p*_ can vary between unconstrained (*M*_*p*_ = 0) to isolated (*M*_*p*_ = 1), providing a level of control over high and low tolerance of false positives respectively. There is evidence that interactions across modules are sparse, while intra-modular connections are dense, suggesting that a good rule of thumb is to favor strong constraints with a higher *M*_*p*_ threshold [70]. Furthermore, SHINE could be extended to include more than the first two eigengenes to account for more variation in the calculation of membership or take advantage of a wide variety of alternative methods for module detection, some of which detect overlapping modules by default [33].

We opted to simulate our own datasets in benchmarking SHINE since existing standardized datasets (e.g., DREAM) were not appropriate for evaluating the key methodological advances presented. First, a salient feature of our approach is the learning of hierarchies of networks; to evaluate this component, we needed to simulate multiple network structures with a tunable level of edge similarity. Second, SHINE is suitable for learning biological networks where distinct co-expression modules can be detected. Thus, we used the LFR benchmark algorithm to simulate modular network structures.

Regarding our choice of existing methods for comparison, as noted, we primarily focused on methods capable of modeling the distinction between direct and indirect dependencies, and thus we deemed ARACNe and GENIE3 to be the most appropriate due to their popularity and wide adoption–which make them gold standards in the field–and their having no requirement for additional data beyond a single observational omics layer, which is the primary use case for SHINE. Additionally, we evaluated five additional GGM-based approaches due to their ability to efficiently model high-dimensional data. Comparison with additional methods was not fruitful due to the large *p/n* ratios included in our evaluation (representative of typical omics data ratios), and/or to the unavailability of readily working code. For example, for the graphical Lasso w/ EBIC tuning [71] (from the R package *qgraph*), ratios greater than 1 should be used, and our application of the algorithm with the low sample sizes we tested (*n* = 20-150/*p* = 300) would immediately fail or hang due to the insufficient number of samples. We also attempted to compare with methods adopting shared learning, such as the joint graphical lasso [29], but attained similar results. While we find SHINE’s focus on single-layer omics data most applicable to the wide availability of such datasets, we acknowledge the drawbacks of this approach. By restricting inference to the transcriptomics layer of a multi-layered biological regulatory network we ignore the interdependencies of genes on other regulatory agents such as proteins, metabolites, non-coding RNAs etc. However, large publicly accessible multi-omics datasets with a strong intersection of samples–where such information could be incorporated into network inference–are rare. When adequately sized multi-omics datasets become available, application of SHINE to their analysis will become feasible.

The learning process is rendered computationally efficient by adoption of the highly optimized core MCMC inference algorithm [72] and, more importantly, by the parallelization made possible by the DAQ approach, as well as by the simultaneous inference of independent networks in a given hierarchy. By using a DAQ approach, the upper bound of *p* scales with the number of sufficiently small modules used as constraints. Thus, while the time complexity of the core inference algorithm remains $O(2^{p^{2}})$, where *p* is the number of genes and *2*^*p(p-1)/2*^ is the possible number of graphs within the search space, the time complexity of SHINE using DAQ is $O(2^{m^{2}})$ where *m* is the number of genes in the largest extended module. In our analysis, we aimed for extended modules smaller than 300 genes, thus using an *M*_*p*_ = 0.9. We believe this is a reasonable limit as over 97% of biological pathways defined in Kegg, Reactome, and Biocarta are smaller than 300 genes. Therefore, SHINE scales well for datasets where a large number of small and tightly clustered co-expression modules can be identified, which in turn depends on the data as well as on the module detection algorithm used. Some datasets may naturally exhibit high levels of correlation and low clustering, resulting in fewer, large modules, which will limit the impact SHINE’s DAQ strategy has on restricting the graphical search space. Similarly, different module detection algorithms will yield differently sized modules. While in this report we focused on WGCNA, we found MEGENA [73] to be a suitable alternative, due to its capability of identifying a larger number of smaller modules. And other algorithms that give more control over the number and size of the modules detected could be considered [33].

Even with the optimization and parallelization strategies we implemented, SHINE is ideally suited for high-performance computing environments. In the analyses presented parallelization was fully automated through adoption of Nextflow to manage network workflows, as implemented in our *shine-nf*. Because some networks are dependent on others (e.g., a network is used as a prior in learning another), *shine-nf* takes advantage of the reactive properties of Nextflow–a language for defining portable, scalable, and reproducible data workflows–to solve and handle the process dependencies between parent and child networks. For example, the application to the Pan-Cancer hierarchy of networks yielded an automatically generated workflow encompassing 656 individual jobs, many of which could be run in parallel, reducing our sequential run time from 52 to 4 hours.

The network hierarchy used to guide shared learning can be specified in advance if it is known, or it can be learned from the data, or a hybrid approach where both “expert knowledge” and a data driven approach to learning can be combined. The use of prior information is highly flexible in that one could incorporate only network constraints, previously learned networks (as a prior distribution of edges), existing network structures (as a seed graph), or a combination.

In our analysis of real datasets from the TCGA, we used a curated set of 1,157 genes. This choice was not motivated by the computational limits of SHINE. Rather, this was the number of genes we extracted from well-characterized cancer pathways, which was our focus in this report. While SHINE is able to learn gene regulatory networks with larger *p*’s, there still exists a tradeoff between network size and accuracy. In practice, it remains advantageous to restrict network analysis to subsets of the genome that a study is primarily interested in. Not only because it will increase accuracy of the resulting networks, but also because it makes the downstream analysis and interpretation of such networks more manageable. We find the analysis, comparison, and most importantly, the visualization and interpretation of multiple biological networks larger than 3–4,000 genes to be challenging, especially at the level of resolution where distinguishing between direct and indirect dependencies remains the main focus.

In summary, we believe SHINE will be a valuable resource for the general reconstruction of biological networks and disease-centric modeling across multiple disciplines. With the increase of large publicly available transcriptomics data for many biological diseases, computational and experimental scientists will be able to model and query disease regulatory network structures to explore and generate hypotheses. While we focus on human gene regulatory networks, it is reasonable to extend this approach to other biological systems and data types, both bulk and single cell data, as well as to other high-throughput omics layers such as proteomics or metabolomics, among others.

## Materials and methods

### Module detection and extension

Genes are clustered by their co-expression similarity *s*_*ij*_−measured by the absolute value of the biweight midcorrelation coefficient: *s*_*i*,*j*_ = | *bicor*(*x*_*i*_, *x*_*j*_) | as well as a soft thresholding value *ß* which pushes spurious correlations to zero, resulting in a symmetric *p x p* weighted adjacency matrix *a*_*ij*_ = *s*_*ij*_^*ß*^. Co-expression modules are detected using hierarchical clustering of a topological overlap dissimilarity transformation *d*_*i*,*j*_ of *a*_*ij*_ resulting in *Q* modules. Genes are assigned a membership score across all modules, where the membership of gene *i* in module *q* is the correlation of *i* and a module eigengene *E*^*(q)*^, which for the *q*^th^ module, is the first principal component of the expression profiles of genes within *q*, thus *MM = | bicor(x*_*i*,_ *E*^*(q)*^*) |*. Within each module, each gene is assigned a probability of membership through quadratic discrimant analysis based on MM1/MM2. Module membership is extended to non-member genes above a membership probability *M*_*p*_.

### Simulation studies

Single network simulations were performed using undirected graphs (*p* = 300) randomly generated using the Lancichinetti–Fortunato–Radicchi (LFR) benchmark (*tau1* = 3, *tau2* = 2, *mu* = 0.08). Specifically, the LFR_benchmark_graph function from the Python package *networkx[74]*. The LFR model generates graphs with overlapping community structures, where both degree and community size follow a power law. The graph structures were used to generate multivariate Gaussian data using the R package *BDgraph* [16] for samples ranging from 20 to 150. Structure learning with constraint-based approaches including Isolated, Extended, and Divide and Conquer (DAQ) was performed for each sample size—repeated 25 times—where a posterior edge probability above 0.9 was considered an edge in the final network. Networks were evaluated based on an F1-score *=* 2TP/(2TP+FP+FN’) where TP, FP, and FN are the number of true positives, false positives, and false negatives respectively and accounts for a balance of detecting TP while limiting FP. Simulations with multiple networks were performed under similar conditions by generating a hierarchy of networks with a known edge similarity. This was done by using an initial graph (*p* = 250) generated by LFR (*tau1* = 3, *tau2* = 2, *mu* = 0.08) to simulate independently diverging network structures (following the rules of preferential attachment via the Barabási–Albert procedure for scale-free network growth)—where divergent networks grow by an additional 25 nodes—arriving at three final graphs (*p* = 300) with an average 84% edge similarity calculated by the Szymkiewicz–Simpson coefficient [75] of edges (see the model.hpa function in the R package *shine*) (Fig 3A). The graph structures were used to generate multivariate Gaussian data using the R package *BDgraph*. Networks were estimated and evaluated with the DAQ constraint method with and without the use of prior network information using previously described methods across increasing samples ranging from 20 to 150—repeated 25 times. Implementations for B_NW_SL, D-S_GL, D-S_NW_SL, GFC_L and GFC_SL were from the R package *SILGGM* (Statistical Inference of Large-Scale Gaussian Graphical Model in Gene Networks) and were used with default settings [76].

### Data Pre-processing

TCGA RNA-Seq count matrices (generated with STAR 2-Pass and HTSeq—Counts) and metadata for all available tumor types was downloaded through the Genomic Data Commons (GDC) *gdc-client* [77]. We performed a variance-stabilizing transformation of the data using the R package *DESeq2* followed by a log-transformation [78]. We removed datasets with fewer than 150 primary tumors, resulting in the following 23 tumor types (L2) and 11 categories (L1): Breast—*BRCA* (*n* = 1102); Thoracic—*LUSC* (*n* = 502), *LUAD* (*n* = 533); Endocrine—*THCA* (*n* = 502), *PCPG* (*n* = 178); Head and Neck—*HNSC* (*n* = 500); CNS—*LGG* (*n* = 511), *GBM* (*n* = 156); Soft Tissue—*SARC* (*n* = 259); Gynecologic—*UCEC* (*n* = 551), *OV* (*n* = 374), *CESC* (*n* = 304); Urologic—*KIRC* (*n* = 538), *PRAD* (*n* = 498), *BLCA* (*n* = 414), *KIRP* (*n* = 288), *TGCT* (*n* = 150); Developmental GI—*LIHC* (*n* = 371), *PAAD* (*n* = 177); Core GI—*COAD* (*n* = 478), *STAD* (*n* = 375), *READ* (*n* = 166), *ESCA* (*n* = 161) (S1 Table). For the Pan-Cancer analysis, gene-wise expression levels were modified by taking the residuals after adjusting for tumor type.

### Gene filtering

Networks were built on genes from the following major cancer pathways sourced from the Molecular Signatures Database (MSigDB) [79] (v7.2.1) and Pathway Commons [80] (v12.0) resulting in a unique set of 1160 genes, 3 of which were not available in the data, further reducing to 1157 final genes. The pathways and geneset sizes include MAPK Signaling (267), Notch Signaling (247), P53 Signaling (200), Jak Stat Signaling (155), DNA Repair (150), TGF-Beta Signaling (54), Oxidative Stress Response (45), PI3K Pathway (43), Wnt/Beta-Catenin Signaling (42), Mtor Signaling (40), Hedge Hog Signaling (36), Myc Signaling (25), RAS Signaling (24), Hippo Signaling (20), Cell Cycle (14) (S2 Table).

### Inference of Pan-Cancer Networks

We computed shared constraints derived from all primary tumors through the R package *shine* which extends methods implemented in the R package *WGCNA* [34]. The soft-thresholding power was set to 3 (the lowest value from 1–20 for which the scale-free topology fit had at least an *R*^*2*^ > 0.80*)*. Co-expression similarity was calculated using biweight midcorrelation and unsigned options. Hierarchical clustering was performed using the average agglomeration method. Adaptive branch pruning was performed using the hybrid method with deep split set to 4 and a minimum cluster size of 10. Lastly, the first module–which represents genes that fail to cluster into a co-expression module of a minimum size–was removed from downstream methods. The resulting 18 modules had sizes ranging from 13–269 with a mean of 63.89 and median of 33. Modules were extended with an *M*_*p*_ = 0.9 extending their sizes to a range of 27–290 with a mean of 82.61 and median of 54. Networks were inferred using the structure learning R package *BDgraph* [16]. We used the undirected GGM search method based on marginal pseudo-likelihood using the birth-death Markov chain Monte Carlo algorithm. Learning constraints were applied with the previously described DAQ strategy. When learning a child network from a parent network, the posterior distribution of edges of the parent was used as the prior in learning the child.

### Inference of breast cancer networks

We computed shared constraints as previously described on all primary breast tumors (*n* = 1102)—DESeq2-log normalized and unadjusted for tumor type—from the following subtype classifications: Luminal A (*n* = 564), Luminal B (*n* = 215), HER2-enriched (*n* = 82), and Basal-like (*n* = 189), Normal-like (*n* = 40), and Indeterminate (*n* = 12). We did not learn an individual network for Normal-like due to its small sample size. The resulting 24 modules had sizes ranging from 11–205 with a mean of 44.38 and median of 28.50. Modules were extended with an *M*_*p*_ = 0.9 extending their sizes to a range of 23–233 with a mean of 68.17 and median of 51.50. Additionally, we used experimentally validated PPIs from MCF-7, MDA-MB-231, MCF-10A, MCF-10AT cell lines annotated in *Federico & Monti* as a structural prior or starting graph in the network hierarchy [59]. Networks were then inferred using the previously described methods.

### Hierarchical workflows

The heavy computational demand for high-dimensional graphical modeling requires networks to be learned in parallel on high performance computing platforms. Because lower-level networks depend on higher-level networks in the hierarchy, we employed the reactive workflow scripting language Nextflow [81] (DSL2) to manage workflows given a network hierarchy within a containerized computing environment via Docker. Together, this enables automatic job parallelization, failure recovery, and portability to various cloud/cluster architectures, resulting in reproducible workflows that are easy to manage. We have developed a collection of Nextflow DSL2 modules for running SHINE as well as a small command line utility written in Python for dynamically generating these workflows based on the hierarchical structure of multiple networks.

### Calculation of network properties and similarity

Networks were formatted into graph objects using the R package *igraph* [82]. We used built-in functions to compute common graph-level and node-level measures such as density, transitivity (global clustering coefficient), clustering (local clustering coefficient), and assortivity. We compared inferred interactions to human experimentally derived protein-protein interactions (PPI) from the Human Integrated Protein-Protein Interaction Reference (HIPPIE)^23^ v2.2. Significance of intersecting sets was computed using a Fischer’s exact test. We also compared the resulting network properties to 1000 iterations (taking the median values) of randomly simulated undirected networks built with the Erdős–Rényi (ER) and Barabási–Albert (BA) models. We computed network similarity or distance via the Kendall rank correlation of nodes ranked by degree for all pairwise networks. Hierarchical clustering was then performed on this distance matrix and visualized as a dendrogram.

### Enrichment of cancer drivers in central nodes

Nodes in each network were ranked by their degree-centrality and tested for enrichment of cancer driver genes identified in *Bailey et*. *al*. in their TCGA Pan-Cancer analysis by a Kolmogorov-Smirnov test [83]. The overall significance was computed by comparing the distribution of p-values arising from each enrichment test to a uniform distribution.

### Computing centrality measures

Degree-centrality for a given node is defined as its number of adjacent edges. Eigen-centrality for a given node, *x*_*i*_, is defined as $\frac{1}{\lambda }\sum_{j=1}^{n}A_{i,j}x_{j}$ where *λ* is a constant, *n* is the total number of nodes in a graph, and *A* is an adjacency matrix with a value of 1 if an edge exists between nodes *i* and *j*, or 0 otherwise[84]. Values are scaled to have a minimum and maximum value of 0 and 1 respectively.

### Computing differential centrality scores

Nodes were ranked by a centrality measure and compared between networks to identify genes differentially connected or central in one or more networks. For *n* nodes, we represent their rank *r* as a Rank Score (RS), computed as *n-r+1* in each network, where a higher rank score indicates higher centrality. RS are weighted (WRS) by an exponent *p* to give more significance to differences between high rankings rather than low rankings where *WRS = rs*^*p*^ and *p* = 25 in the Pan-Cancer analysis. WRS are normalized (NWRS) for each network where *NWRS = wrs/sum(wrs)* within a single network. Using the NWRS, a different centrality score (DCS) for each node is computed as the *max(nwrs)-mean(nwrs)* across networks. We sorted genes by their DCS to find genes differentially central in one or few networks relative to the rest.

### Enrichment of tumor survival genes

We selected the top and bottom 100 genes ranked by their eigen-centrality in each network and visualized their dependency score across various cell lines from the Cancer Dependency Map Project (DepMap) through the R package *depmap* (v1.2); specifically using the CRISPR—Achilles gene effect resource (EH2261) as well as associated cell line metadata (EH2266) [60]. Genes ranked within the top and bottom 100 for each network that were not present in the DepMap were omitted from the analysis. We performed a Wilcoxon test in each network to assess the significance in difference between the distributions of dependency scores for genes within the top and bottom centrality groups.

### Network propagation

We used the random walk with restart (RWR) algorithm, which measures the distance (or proximity) of nodes in a graph from one or multiple seed nodes [64]. It does this by randomly traversing the graph with a given restart probability, starting from the seed node(s). We used the proximity values to visualize the graphical distance of nodes of interest to other nodes in the graph. To characterize the biological function of a set of seed nodes, we ranked traversed nodes by their proximity to the seed nodes(s), which is used as input to the ranked-based Kolmogorov-Smirnov test for enrichment implemented in the R package *hypeR* [85].

### Essential genes signaling pathways

We performed the described network propagation technique (*restart* = 0.5) using the 14 eigen-central genes as seed nodes for each breast cancer subtype network. In Fig 5C we use the parent network (L2) to visualize the network propagation which had a similar global structure to subtype networks (L3). Enrichment was performed using the Reactome curated genesets from MSigDB (v7.2.1) and filtering by FDR < 0.001. Because many of the genesets contain similar gene members and are redundant, we used a hierarchical clustering method based on a pairwise Jaccard index to selectively highlight non-redundant genesets enriched across multiple networks [86].

### Biological pathway enrichment

Enrichment of biological processes and pathways was performed with the R package *hypeR*. We used both the hypergeometric test for overrepresentation and the ranked-based Kolmogorov-Smirnov test for enrichment. We used curated genesets from MSigDB including v7.2.1 of the Hallmark and Reactome collections.

### Disease neighborhoods

We performed community detection on networks using the Walktrap [65] algorithm (*steps* = 10). Communities were tested for enrichment of known breast cancer disease genes via a hypergeometric test as previously described and using expert curated breast carcinoma disease genes (C0678222) from DisGeNET [66] (v7.0). Identified disease neighborhoods were tested for enrichment of biological pathways using the hypergeometric test for overrepresentation with the R package *hypeR*. Enrichment was performed using the Hallmark curated genesets from MSigDB (v7.2.1) and filtering by FDR < 0.05. Guilt by association with breast cancer disease genes was computed by performing network propagation within each disease neighborhood, using disease genes as seed nodes as previously described (*restart* = 0.7). Druggable targets were identified from The Genomics of Drug Sensitivity in Cancer [67].

License: GNU GPLv3

## Supporting information

S1 Fig Pseudocode for Learning Hierarchical Networks. The learning procedure is carried out in Nextflow and depends on a workflow file describing the hierarchical structure of the networks to be learned. Presented is a basic example of learning one parent and one child network (A and B respectively) that can be expanded to accommodate more complex hierarchical structures. The pseudocode describes the DAQ strategy, a 2-step procedure whereby each module is learned independently and the final network is reconstructed from the resulting subgraphs. In this example, two expressions sets are provided, one for each group of samples the networks are to be learned for (A and B). An additional requirement is one or more modules of genes to serve as shared constraints. Firstly, network A subgraphs are learned with an uninformative prior for each module of genes (LEARN). Network A is reconstructed from these subgraphs, conserving edges present in all local conditions (subgraphs) where they are tested (RECONSTRUCT). In learning network B subgraphs, the corresponding previously outputted (LearnNetworkA.out) network A subgraphs are used as priors (LEARN_PRIOR).
(DOCX)

S2 Fig SHINE Outperforms at Low Sample Sizes in Recapturing Experimentally Validated Interactions. SHINE was compared to GFC_SL on TCGA Pan-Cancer data to reconstruct networks for 1,157 genes across 23 tumor types. Inferred network edges by both methods were tested against a database of experimentally validated PPIs in human. SHINE outperforms on 20/23 networks, and performs particularly well when the number of available samples (*n*) is small compared to the number of nodes learned. TCGA study names for network abbreviations can be found in the S1 Appendix.
(DOCX)

S3 Fig Hierarchical Learning of TCGA Pan-Cancer Networks. Networks were learned using extended modules constructed on L0 data. These constraints were shared to learn L1 networks using the DAQ approach. L2 networks were learned using L1 as a prior based on the TCGA pre-defined hierarchy.
(SVG)

S4 Fig Hierarchical Learning of TCGA Breast Cancer Networks. Networks were learned using extended modules constructed at the root level of the TCGA-BRCA hierarchy. Interactions from experimentally validated PPIs within the defined constraints were used as a structural prior or starting graph in the network hierarchy. The structural prior was used to learn the L2 network using the DAQ approach. L3 networks were learned using L2 as a prior.
(SVG)

S5 Fig Confirmation of siRNA Silencing by RT-qPCR. RT-qPCR analysis showing relative expression of genes silenced by siRNA in MDA-MB-231 cells (n = 3. Unpaired t-test. Data are shown as mean ± SEM. ***p≤0.001, ****p≤0.0001).
(DOCX)

S1 Table Data Sample Sizes. This file reports the tumor type categories and their respective sample size.
(XLSX)

S2 Table Major Cancer Pathways. This file reports the major cancer pathways used in sourcing genes for network learning.
(XLSX)

S3 Table SYBR RT-qPCR Primers. This table lists the primers used for RT-qPCR experiments shown in S5 Fig.
(DOCX)

S1 Appendix TCGA Pan-Cancer Network Abbreviations. (DOCX)

## References

[1] Barabási A-L, Gulbahce N, Loscalzo J. Network medicine: a network-based approach to human disease. Nat Rev Genet. 2011;12: 56–68. doi: 10.1038/nrg2918 21164525 PMC3140052

[2] Vidal M, Cusick ME, Barabási A-L. Interactome Networks and Human Disease. Cell. 2011;144: 986–998. doi: 10.1016/j.cell.2011.02.016 21414488 PMC3102045

[3] Huttlin EL, Bruckner RJ, Paulo JA, Cannon JR, Ting L, Baltier K, et al. Architecture of the human interactome defines protein communities and disease networks. Nature. 2017;545: 505–509. doi: 10.1038/nature22366 28514442 PMC5531611

[4] van Dam S, Võsa U, van der Graaf A, Franke L, de Magalhães JP. Gene co-expression analysis for functional classification and gene–disease predictions. Brief Bioinform. 2018;19: 575–592. doi: 10.1093/bib/bbw139 28077403 PMC6054162

[5] Zhang B, Gaiteri C, Bodea L-G, Wang Z, McElwee J, Podtelezhnikov AA, et al. Integrated systems approach identifies genetic nodes and networks in late-onset Alzheimer’s disease. Cell. 2013;153: 707–720. doi: 10.1016/j.cell.2013.03.030 23622250 PMC3677161

[6] Anglani R, Creanza TM, Liuzzi VC, Piepoli A, Panza A, Andriulli A, et al. Loss of Connectivity in Cancer Co-Expression Networks. PLOS ONE. 2014;9: e87075. doi: 10.1371/journal.pone.0087075 24489837 PMC3904972

[7] Brænne I, Onengut-Gumuscu S, Chen R, Manichaikul AW, Rich SS, Chen W-M, et al. Dynamic changes in immune gene co-expression networks predict development of type 1 diabetes. Sci Rep. 2021;11: 22651. doi: 10.1038/s41598-021-01840-z 34811390 PMC8609030

[8] Friedman N, Linial M, Nachman I, Pe’er D. Using Bayesian Networks to Analyze Expression Data. J Comput Biol. 2000;7: 601–620. doi: 10.1089/106652700750050961 11108481

[9] Friedman N. Inferring Cellular Networks Using Probabilistic Graphical Models. Science. 2004;303: 799–805. doi: 10.1126/science.1094068 14764868

[10] Feizi S, Marbach D, Médard M, Kellis M. Network deconvolution as a general method to distinguish direct dependencies in networks. Nat Biotechnol. 2013;31: 726–733. doi: 10.1038/nbt.2635 23851448 PMC3773370

[11] Friedman J, Hastie T, Tibshirani R. Sparse inverse covariance estimation with the graphical lasso. Biostatistics. 2008;9: 432–441. doi: 10.1093/biostatistics/kxm045 18079126 PMC3019769

[12] Huynh-Thu VA, Irrthum A, Wehenkel L, Geurts P. Inferring Regulatory Networks from Expression Data Using Tree-Based Methods. PLOS ONE. 2010;5: e12776. doi: 10.1371/journal.pone.0012776 20927193 PMC2946910

[13] de la Fuente A, Bing N, Hoeschele I, Mendes P. Discovery of meaningful associations in genomic data using partial correlation coefficients. Bioinformatics. 2004;20: 3565–3574. doi: 10.1093/bioinformatics/bth445 15284096

[14] Witten DM, Friedman JH, Simon N. New Insights and Faster Computations for the Graphical Lasso. J Comput Graph Stat. 2011;20: 892–900. doi: 10.1198/jcgs.2011.11051a

[15] Peterson C, Stingo FC, Vannucci M. Bayesian Inference of Multiple Gaussian Graphical Models. J Am Stat Assoc. 2015;110: 159–174. doi: 10.1080/01621459.2014.896806 26078481 PMC4465207

[16] Mohammadi R, Wit EC. BDgraph: An R Package for Bayesian Structure Learning in Graphical Models. J Stat Softw. 2019;89: 1–30. doi: 10.18637/jss.v089.i03

[17] Ren Z, Sun T, Zhang C-H, Zhou HH. Asymptotic normality and optimalities in estimation of large Gaussian graphical models. Ann Stat. 2015;43: 991–1026. doi: 10.1214/14-AOS1286

[18] Janková J, Geer S van de. Confidence intervals for high-dimensional inverse covariance estimation. Electron J Stat. 2015;9: 1205–1229. doi: 10.1214/15-EJS1031

[19] Janková J, Geer S. Honest confidence regions and optimality in high-dimensional precision matrix estimation. TEST Off J Span Soc Stat Oper Res. 2017;26: 143–162.

[20] Liu W. Gaussian graphical model estimation with false discovery rate control. Ann Stat. 2013;41: 2948–2978. doi: 10.1214/13-AOS1169

[21] Marbach D, Costello JC, Küffner R, Vega NM, Prill RJ, Camacho DM, et al. Wisdom of crowds for robust gene network inference. Nat Methods. 2012;9: 796–804. doi: 10.1038/nmeth.2016 22796662 PMC3512113

[22] West M. Bayesian Factor Regression Models in the “Large p, Small n” Paradigm. Bayesian Statistics. Oxford University Press; 2003. pp. 723–732.

[23] Johnstone IM, Titterington DM. Statistical challenges of high-dimensional data. Philos Trans R Soc Math Phys Eng Sci. 2009;367: 4237–4253. doi: 10.1098/rsta.2009.0159 19805443 PMC2865881

[24] Pensar J, Nyman H, Niiranen J, Corander J. Marginal Pseudo-Likelihood Learning of Discrete Markov Network Structures. Bayesian Anal. 2017;12: 1195–1215. doi: 10.1214/16-BA1032

[25] Segal E, Shapira M, Regev A, Pe’er D, Botstein D, Koller D, et al. Module networks: identifying regulatory modules and their condition-specific regulators from gene expression data. Nat Genet. 2003;34: 166–176. doi: 10.1038/ng1165 12740579

[26] Stuart JM, Segal E, Koller D, Kim SK. A gene-coexpression network for global discovery of conserved genetic modules. Science. 2003;302: 249–255. doi: 10.1126/science.1087447 12934013

[27] Pierson E, Consortium the Gte, Koller D, Battle A, Mostafavi S. Sharing and Specificity of Co-expression Networks across 35 Human Tissues. PLOS Comput Biol. 2015;11: e1004220. doi: 10.1371/journal.pcbi.1004220 25970446 PMC4430528

[28] Omranian N, Eloundou-Mbebi JMO, Mueller-Roeber B, Nikoloski Z. Gene regulatory network inference using fused LASSO on multiple data sets. Sci Rep. 2016;6: 20533. doi: 10.1038/srep20533 26864687 PMC4750075

[29] Danaher P, Wang P, Witten DM. The joint graphical lasso for inverse covariance estimation across multiple classes. J R Stat Soc Ser B Stat Methodol. 2014;76: 373–397. doi: 10.1111/rssb.12033 24817823 PMC4012833

[30] Hartwell LH, Hopfield JJ, Leibler S, Murray AW. From molecular to modular cell biology. Nature. 1999;402: C47–C52. doi: 10.1038/35011540 10591225

[31] Ravasz E, Barabási A-L. Hierarchical organization in complex networks. Phys Rev E. 2003;67: 026112. doi: 10.1103/PhysRevE.67.026112 12636753

[32] Zhao J, Ding G-H, Tao L, Yu H, Yu Z-H, Luo J-H, et al. Modular co-evolution of metabolic networks. BMC Bioinformatics. 2007;8: 311. doi: 10.1186/1471-2105-8-311 17723146 PMC2001200

[33] Saelens W, Cannoodt R, Saeys Y. A comprehensive evaluation of module detection methods for gene expression data. Nat Commun. 2018;9: 1090. doi: 10.1038/s41467-018-03424-4 29545622 PMC5854612

[34] Langfelder P, Horvath S. WGCNA: an R package for weighted correlation network analysis. BMC Bioinformatics. 2008;9: 559. doi: 10.1186/1471-2105-9-559 19114008 PMC2631488

[35] Dong J, Horvath S. Understanding network concepts in modules. BMC Syst Biol. 2007;1: 24. doi: 10.1186/1752-0509-1-24 17547772 PMC3238286

[36] Song L, Langfelder P, Horvath S. Comparison of co-expression measures: mutual information, correlation, and model based indices. BMC Bioinformatics. 2012;13: 328. doi: 10.1186/1471-2105-13-328 23217028 PMC3586947

[37] Kitano H. Computational systems biology. Nature. 2002;420: 206–210. doi: 10.1038/nature01254 12432404

[38] Lancichinetti A, Fortunato S, Radicchi F. Benchmark graphs for testing community detection algorithms. Phys Rev E. 2008;78: 046110. doi: 10.1103/PhysRevE.78.046110 18999496

[39] Barabási A-L, Albert R. Emergence of Scaling in Random Networks. Science. 1999;286: 509–512. doi: 10.1126/science.286.5439.509 10521342

[40] Margolin AA, Nemenman I, Basso K, Wiggins C, Stolovitzky G, Favera RD, et al. ARACNE: An Algorithm for the Reconstruction of Gene Regulatory Networks in a Mammalian Cellular Context. BMC Bioinformatics. 2006;7: S7. doi: 10.1186/1471-2105-7-S1-S7 16723010 PMC1810318

[41] Califano A, Alvarez MJ. The recurrent architecture of tumour initiation, progression and drug sensitivity. Nat Rev Cancer. 2017;17: 116–130. doi: 10.1038/nrc.2016.124 27977008 PMC5541669

[42] Sanchez-Vega F, Mina M, Armenia J, Chatila WK, Luna A, La KC, et al. Oncogenic Signaling Pathways in The Cancer Genome Atlas. Cell. 2018;173: 321–337.e10. doi: 10.1016/j.cell.2018.03.035 29625050 PMC6070353

[43] Sever R, Brugge JS. Signal Transduction in Cancer. Cold Spring Harb Perspect Med. 2015;5: a006098. doi: 10.1101/cshperspect.a006098 25833940 PMC4382731

[44] Erdös P, Renyi A. On the Strength of Connectedness of a Random Graph. 1961.

[45] Alanis-Lobato G, Andrade-Navarro MA, Schaefer MH. HIPPIE v2.0: enhancing meaningfulness and reliability of protein–protein interaction networks. Nucleic Acids Res. 2017;45: D408–D414. doi: 10.1093/nar/gkw985 27794551 PMC5210659

[46] Networks Newman M. Second Edition. Oxford, New York: Oxford University Press; 2018.

[47] Wang Q, Pan F, Li S, Huang R, Wang X, Wang S, et al. The prognostic value of the proteasome activator subunit gene family in skin cutaneous melanoma. J Cancer. 2019;10: 2205–2219. doi: 10.7150/jca.30612 31258724 PMC6584401

[48] Wang S, Ren T, Jiao G, Huang Y, Bao X, Zhang F, et al. BMPR2 promotes invasion and metastasis via the RhoA-ROCK-LIMK2 pathway in human osteosarcoma cells. Oncotarget. 2017;8: 58625–58641. doi: 10.18632/oncotarget.17382 28938584 PMC5601680

[49] Xia S, Tang Q, Wang X, Zhang L, Jia L, Wu D, et al. Overexpression of PSMA7 predicts poor prognosis in patients with gastric cancer. Oncol Lett. 2019;18: 5341–5349. doi: 10.3892/ol.2019.10879 31612044 PMC6781669

[50] McCorkle JR, Leonard MK, Kraner SD, Blalock EM, Ma D, Zimmer SG, et al. The metastasis suppressor NME1 regulates expression of genes linked to metastasis and patient outcome in melanoma and breast carcinoma. Cancer Genomics Proteomics. 2014;11: 175–194. 25048347 PMC4409327

[51] Filipek PA, de Araujo MEG, Vogel GF, De Smet CH, Eberharter D, Rebsamen M, et al. LAMTOR/Ragulator is a negative regulator of Arl8b- and BORC-dependent late endosomal positioning. J Cell Biol. 2017;216: 4199–4215. doi: 10.1083/jcb.201703061 28993467 PMC5716276

[52] Gayther SA, Batley SJ, Linger L, Bannister A, Thorpe K, Chin S-F, et al. Mutations truncating the EP300 acetylase in human cancers. Nat Genet. 2000;24: 300–303. doi: 10.1038/73536 10700188

[53] Pickering CR, Zhou JH, Lee JJ, Drummond JA, Peng SA, Saade RE, et al. Mutational landscape of aggressive cutaneous squamous cell carcinoma. Clin Cancer Res Off J Am Assoc Cancer Res. 2014;20: 6582–6592. doi: 10.1158/1078-0432.CCR-14-1768 25303977 PMC4367811

[54] Jouenne F, Reger de Moura C, Lorillon G, Meignin V, Dumaz N, Lebbe C, et al. RASA1 loss in a BRAF-mutated Langerhans cell sarcoma: a mechanism of resistance to BRAF inhibitor. Ann Oncol. 2019;30: 1170–1172. doi: 10.1093/annonc/mdz125 30977771

[55] Shen S, Dean DC, Yu Z, Duan Z. Role of cyclin-dependent kinases (CDKs) in hepatocellular carcinoma: Therapeutic potential of targeting the CDK signaling pathway. Hepatol Res. 2019;49: 1097–1108. doi: 10.1111/hepr.13353 31009153

[56] Lo J-F, Yu C-C, Chiou S-H, Huang C-Y, Jan C-I, Lin S-C, et al. The Epithelial-Mesenchymal Transition Mediator S100A4 Maintains Cancer-Initiating Cells in Head and Neck Cancers. Cancer Res. 2011;71: 1912–1923. doi: 10.1158/0008-5472.CAN-10-2350 21169409

[57] Fotovati A, Abu-Ali S, Wang P-S, Deleyrolle LP, Lee C, Triscott J, et al. YB-1 Bridges Neural Stem Cells and Brain Tumor–Initiating Cells via Its Roles in Differentiation and Cell Growth. Cancer Res. 2011;71: 5569–5578. doi: 10.1158/0008-5472.CAN-10-2805 21730024

[58] Berger AC, Korkut A, Kanchi RS, Hegde AM, Lenoir W, Liu W, et al. A Comprehensive Pan-Cancer Molecular Study of Gynecologic and Breast Cancers. Cancer Cell. 2018;33: 690–705.e9. doi: 10.1016/j.ccell.2018.03.014 29622464 PMC5959730

[59] Federico A, Monti S. Contextualized Protein-Protein Interactions. Patterns. 2021;2: 100153. doi: 10.1016/j.patter.2020.100153 33511361 PMC7815950

[60] Tsherniak A, Vazquez F, Montgomery PG, Weir BA, Kryukov G, Cowley GS, et al. Defining a Cancer Dependency Map. Cell. 2017;170: 564–576.e16. doi: 10.1016/j.cell.2017.06.010 28753430 PMC5667678

[61] Ruhnau B. Eigenvector-centrality—a node-centrality? Soc Netw. 2000;22: 357–365. doi: 10.1016/S0378-8733(00)00031-9

[62] Gysi DM, Valle Í do, Zitnik M, Ameli A, Gan X, Varol O, et al. Network medicine framework for identifying drug-repurposing opportunities for COVID-19. Proc Natl Acad Sci. 2021;118. doi: 10.1073/pnas.2025581118 33906951 PMC8126852

[63] Tong H, Faloutsos C, Pan J. Fast Random Walk with Restart and Its Applications. Sixth International Conference on Data Mining (ICDM’06). 2006. pp. 613–622. doi: 10.1109/ICDM.2006.70

[64] Macropol K, Can T, Singh AK. RRW: repeated random walks on genome-scale protein networks for local cluster discovery. BMC Bioinformatics. 2009;10: 283. doi: 10.1186/1471-2105-10-283 19740439 PMC2748087

[65] Pons P, Latapy M. Computing communities in large networks using random walks. arXiv:physics/0512106. 2005 [cited 24 May 2021]. Available: http://arxiv.org/abs/physics/0512106.

[66] Piñero J, Bravo À, Queralt-Rosinach N, Gutiérrez-Sacristán A, Deu-Pons J, Centeno E, et al. DisGeNET: a comprehensive platform integrating information on human disease-associated genes and variants. Nucleic Acids Res. 2017;45: D833–D839. doi: 10.1093/nar/gkw943 27924018 PMC5210640

[67] Yang W, Soares J, Greninger P, Edelman EJ, Lightfoot H, Forbes S, et al. Genomics of Drug Sensitivity in Cancer (GDSC): a resource for therapeutic biomarker discovery in cancer cells. Nucleic Acids Res. 2013;41: D955–D961. doi: 10.1093/nar/gks1111 23180760 PMC3531057

[68] Papadopoulos N, Lennartsson J. The PDGF/PDGFR pathway as a drug target. Mol Aspects Med. 2018;62: 75–88. doi: 10.1016/j.mam.2017.11.007 29137923

[69] Jansson S, Aaltonen K, Bendahl P-O, Falck A-K, Karlsson M, Pietras K, et al. The PDGF pathway in breast cancer is linked to tumour aggressiveness, triple-negative subtype and early recurrence. Breast Cancer Res Treat. 2018;169: 231–241. doi: 10.1007/s10549-018-4664-7 29380207 PMC5945746

[70] Wang Z, Zhang J. In Search of the Biological Significance of Modular Structures in Protein Networks. PLOS Comput Biol. 2007;3: e107. doi: 10.1371/journal.pcbi.0030107 17542644 PMC1885274

[71] Foygel R, Drton M. Extended Bayesian Information Criteria for Gaussian Graphical Models. Advances in Neural Information Processing Systems. Curran Associates, Inc.; 2010. Available: https://papers.nips.cc/paper/2010/hash/072b030ba126b2f4b2374f342be9ed44-Abstract.html.

[72] Mohammadi R, Massam H, Letac G. Accelerating Bayesian Structure Learning in Sparse Gaussian Graphical Models. J Am Stat Assoc. 2021;0: 1–14. doi: 10.1080/01621459.2021.1996377

[73] Song W-M, Zhang B. Multiscale Embedded Gene Co-expression Network Analysis. PLOS Comput Biol. 2015;11: e1004574. doi: 10.1371/journal.pcbi.1004574 26618778 PMC4664553

[74] Hagberg AA, Schult DA, Swart PJ. Exploring Network Structure, Dynamics, and Function using NetworkX. 2008; 5.

[75] K V M., K K A Survey on Similarity Measures in Text Mining. Mach Learn Appl Int J. 2016;3: 19–28. doi: 10.5121/mlaij.2016.3103

[76] Zhang R, Ren Z, Chen W. SILGGM: An extensive R package for efficient statistical inference in large-scale gene networks. PLOS Comput Biol. 2018;14: e1006369. doi: 10.1371/journal.pcbi.1006369 30102702 PMC6107288

[77] Grossman RL, Heath AP, Ferretti V, Varmus HE, Lowy DR, Kibbe WA, et al. Toward a Shared Vision for Cancer Genomic Data. N Engl J Med. 2016;375: 1109–1112. doi: 10.1056/NEJMp1607591 27653561 PMC6309165

[78] Love MI, Huber W, Anders S. Moderated estimation of fold change and dispersion for RNA-seq data with DESeq2. Genome Biol. 2014;15: 550. doi: 10.1186/s13059-014-0550-8 25516281 PMC4302049

[79] Liberzon A, Subramanian A, Pinchback R, Thorvaldsdóttir H, Tamayo P, Mesirov JP. Molecular signatures database (MSigDB) 3.0. Bioinformatics. 2011;27: 1739–1740. doi: 10.1093/bioinformatics/btr260 21546393 PMC3106198

[80] Cerami EG, Gross BE, Demir E, Rodchenkov I, Babur Ö, Anwar N, et al. Pathway Commons, a web resource for biological pathway data. Nucleic Acids Res. 2011;39: D685–D690. doi: 10.1093/nar/gkq1039 21071392 PMC3013659

[81] Di Tommaso P, Chatzou M, Floden EW, Barja PP, Palumbo E, Notredame C. Nextflow enables reproducible computational workflows. Nat Biotechnol. 2017;35: 316–319. doi: 10.1038/nbt.3820 28398311

[82] Csardi G, Nepusz T. The igraph software package for complex network research. InterJournal Complex Syst. 2006; 9.

[83] Bailey MH, Tokheim C, Porta-Pardo E, Sengupta S, Bertrand D, Weerasinghe A, et al. Comprehensive Characterization of Cancer Driver Genes and Mutations. Cell. 2018;173: 371–385.e18. doi: 10.1016/j.cell.2018.02.060 29625053 PMC6029450

[84] Newman MEJ. Mathematics of Networks. The New Palgrave Dictionary of Economics. London: Palgrave Macmillan UK; 2016. pp. 1–8. doi: 10.1057/978-1-349-95121-5_2565–1

[85] Federico A, Monti S. hypeR: an R package for geneset enrichment workflows. Bioinformatics. 2020;36: 1307–1308. doi: 10.1093/bioinformatics/btz700 31498385 PMC7998712

[86] Pedersen TL. Hierarchical sets: analyzing pangenome structure through scalable set visualizations. Bioinformatics. 2017;33: 1604–1612. doi: 10.1093/bioinformatics/btx034 28130242 PMC5447240

