# Supplementary material for: Structure learning for gene regulatory networks
Source: PLoS Comput Biol. 2023 May 18;19(5):e1011118. doi: 10.1371/journal.pcbi.1011118 (PMC10231840; doi:10.1371/journal.pcbi.1011118)
Supplement: S1 Fig — The learning procedure is carried out in Nextflow and depends on a workflow file describing the hierarchical structure of the networks to be learned. Presented is a basic example of learning one parent and one child network (A and B respectively) that can be expanded to accommodate more complex hierarchical structures. The pseudocode describes the DAQ strategy, a 2-step procedure whereby each module is learned independently and the final network is reconstructed from the resulting subgraphs. In this example, two expressions sets are provided, one for each group of samples the networks are to be learned for (A and B). An additional requirement is one or more modules of genes to serve as shared constraints. Firstly, network A subgraphs are learned with an uninformative prior for each module of genes (LEARN). Network A is reconstructed from these subgraphs, conserving edges present in all local conditions (subgraphs) where they are tested (RECONSTRUCT). In learning network B subgraphs, the corresponding previously outputted (LearnNetworkA.out) network A subgraphs are used as priors (LEARN_PRIOR). (DOCX) [file pcbi.1011118.s001.docx]

**Supplementary Figure S1: Pseudocode for learning hierarchical networks**

The learning procedure is carried out in Nextflow and depends on a workflow file describing the hierarchical structure of the networks to be learned. Presented is a basic example of learning one parent and one child network (A and B respectively) that can be expanded to accommodate more complex hierarchical structures. The pseudocode describes the DAQ strategy, a 2-step procedure whereby each module is learned independently and the final network is reconstructed from the resulting subgraphs. In this example, two expressions sets are provided, one for each group of samples the networks are to be learned for (A and B). An additional requirement is one or more modules of genes to serve as shared constraints. Firstly, network A subgraphs are learned with an uninformative prior for each module of genes (LEARN). Network A is reconstructed from these subgraphs, conserving edges present in all local conditions (subgraphs) where they are tested (RECONSTRUCT). In learning network B subgraphs, the corresponding previously outputted (LearnNetworkA.out) network A subgraphs are used as priors (LEARN_PRIOR).
