# Supplementary material for: Structure learning for gene regulatory networks
Source: PLoS Comput Biol. 2023 May 18;19(5):e1011118. doi: 10.1371/journal.pcbi.1011118 (PMC10231840; doi:10.1371/journal.pcbi.1011118)
Supplement: S2 Fig — SHINE was compared to GFC_SL on TCGA Pan-Cancer data to reconstruct networks for 1,157 genes across 23 tumor types. Inferred network edges by both methods were tested against a database of experimentally validated PPIs in human. SHINE outperforms on 20/23 networks, and performs particularly well when the number of available samples (n) is small compared to the number of nodes learned. TCGA study names for network abbreviations can be found in the S1 Appendix. (DOCX) [file pcbi.1011118.s002.docx]

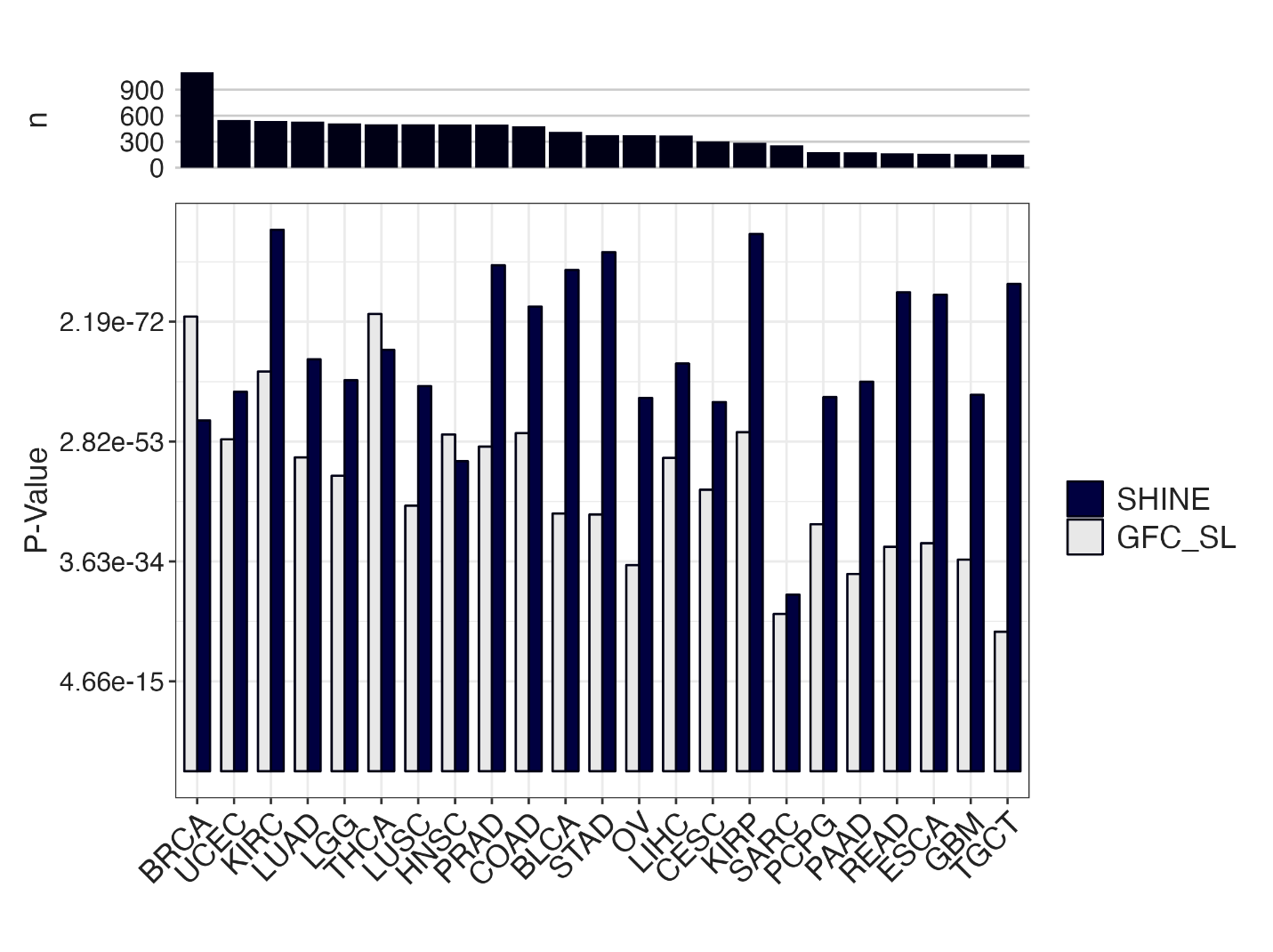


**S2 Fig. SHINE Outperforms at Low Sample Sizes in Recapturing Experimentally Validated Interactions.**

SHINE was compared to GFC_SL on TCGA Pan-Cancer data to reconstruct networks for 1,157 genes across 23 tumor types. Inferred network edges by both methods were tested against a database of experimentally validated PPIs in human. SHINE outperforms on 20/23 networks, and performs particularly well when the number of available samples (*n*) is small compared to the number of nodes learned. TCGA study names for network abbreviations can be found in the S1 Appendix.
