# Supplementary material for: Structure learning for gene regulatory networks
Source: PLoS Comput Biol. 2023 May 18;19(5):e1011118. doi: 10.1371/journal.pcbi.1011118 (PMC10231840; doi:10.1371/journal.pcbi.1011118)
Supplement: S5 Fig — RT-qPCR analysis showing relative expression of genes silenced by siRNA in MDA-MB-231 cells (n = 3. Unpaired t-test. Data are shown as mean ± SEM. ***p≤0.001, ****p≤0.0001). (DOCX) [file pcbi.1011118.s005.docx]

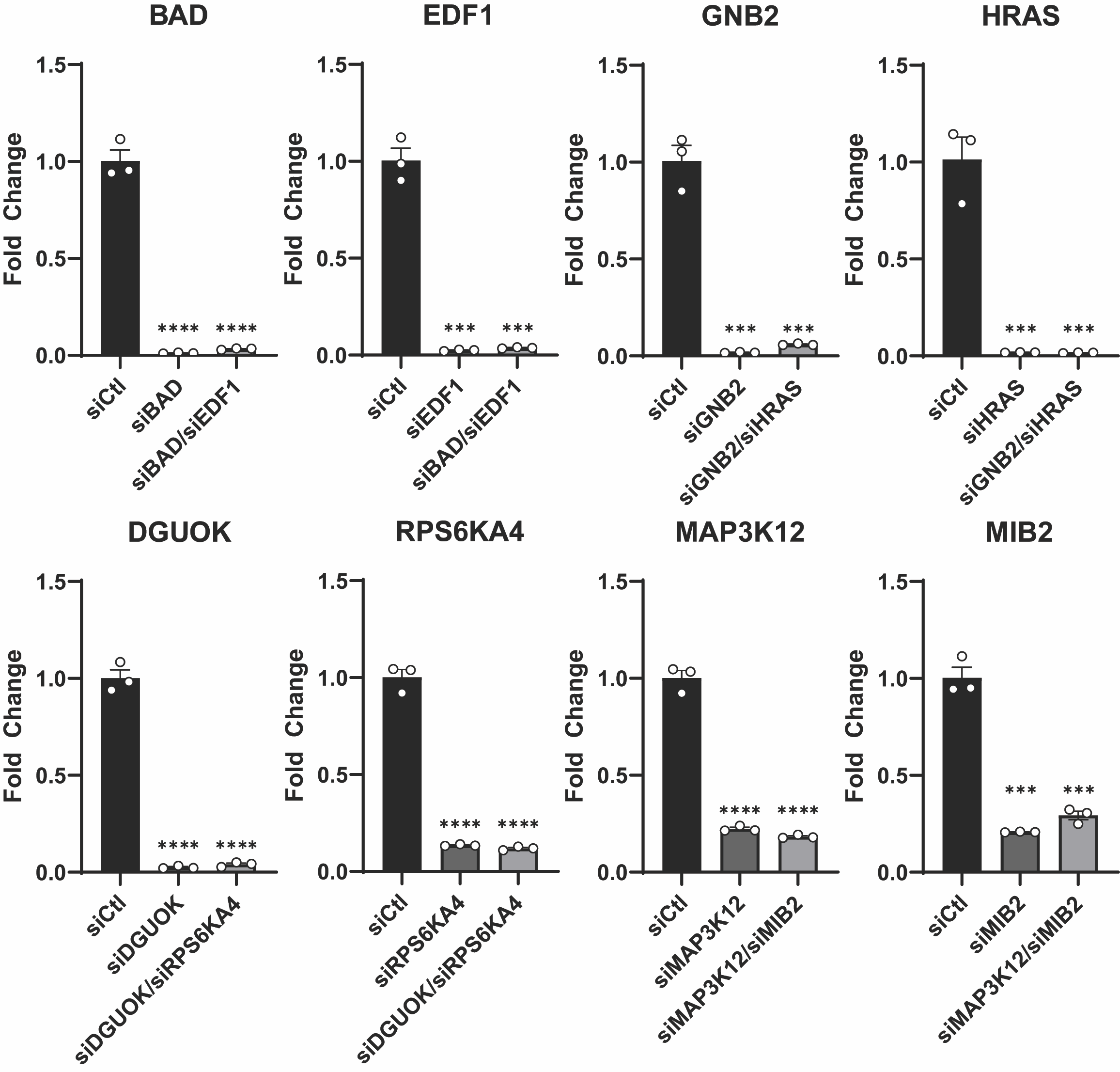


**Supplementary Figure S5: Confirmation of siRNA Silencing by RT-qPCR**

RT-qPCR analysis showing relative expression of genes silenced by siRNA in MDA-MB-231 cells (n=3. Unpaired t-test. Data are shown as mean ± SEM. ***p≤0.001, ****p≤0.0001).
