# Supplementary material for: Structure learning for gene regulatory networks
Source: PLoS Comput Biol. 2023 May 18;19(5):e1011118. doi: 10.1371/journal.pcbi.1011118 (PMC10231840; doi:10.1371/journal.pcbi.1011118)
Supplement: S3 Table — This table lists the primers used for RT-qPCR experiments shown in S5 Fig. (DOCX) [file pcbi.1011118.s008.docx]

**Supplementary Table S3: SYBR RT-qPCR Primers**

| **Target** | **Sequence** |
| --- | --- |
| BAD-F | CGAGTTTGTGGACTCCTTTAAGA |
| BAD-R | CACCAGGACTGGAAGACTCG |
| EDF1-F | AGGTGATCGCGGACTATGAG |
| EDF1-R | GGGCTTTCCAATGTCCTTTC |
| GNB2-F | TTCCTGGATGACAACCAAATC |
| GNB2-R | AGCAAAACCCACTGTCTGCT |
| HRAS-F | GCACGCACTGTGGAATCTC |
| HRAS-R | TAGAAGGCATCCTCCACTCC |
| DGUOK-F | TTTCTCCTGTGGGAGTTTGC |
| DGUOK-R | CTGGCCCTCTGGTACAGTCT |
| RPS6KA4-F | GGCTGGAGCCTGTCTACTCA |
| RPS6KA4-R | GCGTTGTTGTGGTCAAAGAG |
| MAP3K12-F | GGTGTGGGAAGCAACAGTCT |
| MAP3K12-R | GCGATTTCGTGGTTTGCTAT |
| MIB2-F | GGGGACAAGGTCAAGTGTCT |
| MIB2-R | CCGTCTGTCCGATAAACTCC |
| GAPDH-F | AATCCCATCACCATCTTCCA |
| GAPDH-R | TGGACTCCACGACGTACTCA |
