# Supplementary material for: Structure learning for gene regulatory networks
Source: PLoS Comput Biol. 2023 May 18;19(5):e1011118. doi: 10.1371/journal.pcbi.1011118 (PMC10231840; doi:10.1371/journal.pcbi.1011118)
Supplement: S1 Appendix — (DOCX) [file pcbi.1011118.s009.docx]

**S1 Appendix**

**TCGA Pan-Cancer Network Abbreviations**BLCA - Bladder Urothelial Carcinoma

BRCA - Breast Invasive Carcinoma

CESC - Cervical Squamous Cell Carcinoma and Endocervical Adenocarcinoma

COAD - Colon Adenocarcinoma

ESCA - Esophageal Carcinoma

GBM - Glioblastoma Multiforme

HNSC - Head and Neck Squamous Cell Carcinoma

KIRC - Kidney Renal Clear Cell Carcinoma

KIRP - Kidney Renal Papillary Cell Carcinoma

LGG - Brain Lower Grade Glioma

LIHC - Liver Hepatocellular Carcinoma

LUAD - Lung Adenocarcinoma

LUSC - Lung Squamous Cell Carcinoma

OV - Ovarian Serous Cystadenocarcinoma

PAAD - Pancreatic Adenocarcinoma

PCPG - Pheochromocytoma and Paraganglioma

PRAD - Prostate Adenocarcinoma

READ - Rectum Adenocarcinoma

SARC - Sarcoma

STAD - Stomach Adenocarcinoma

TGCT - Testicular Germ Cell Tumors

THCA - Thyroid Carcinoma

UCEC - Uterine Corpus Endometrial Carcinoma

**Validation of Breast Cancer Essential Genes**

We found 14 genes - *BAD*, *DGUOK*, *EDF1*, *GNB2*, *HBP1*, *HRAS*, *MAP3K12*, *MIB2*, *PIDD1*, *POLR2J*, *PSMC1*, *RFC4*, *RPS6KA4*, *TAF1C* - to be ranked within the top 25 by eigen-centrality in all four breast cancer subtype networks. We used dependency scores across various cell lines from the DepMap; specifically using the CRISPR - Achilles gene effect resource (EH2261) as well as associated cell line metadata (EH2266). Genes with an average dependency score > -0.5 across cell lines were considered not to have been previously identified as essential to tumor survival. We used the R package *hierarchicalSets* (v1.0.3) to identify likely gene pairs based on intersecting sets of interactors in the parent breast cancer network (L2). We performed cell viability assays on pairs where both genes were not previously found to be essential to tumor survival by the DepMap. The significance of relative viability after knockdown was computed with a one-sided t-test (*n = 6*).

**Cell Culture**

MDA-MB-231 cells were cultured in Dulbecco’s Modified Eagle’s Medium (Corning, 10-013-CV) supplemented with 10% fetal bovine serum (Gibco, 26140), 100 units/mL penicillin, and 100 µg/mL streptomycin (Gibco, 15140). Cells were cultured in media without antibiotics when performing siRNA transfections.

**Cell Viability Assay**

MDA-MB-231 cells were seeded at a concentration of 2,500 cells/well in a 96-well format and reverse transfected with the indicated siRNAs using Lipofectamine RNAiMAX (Invitrogen, 13778075) according to the manufacturer’s instructions. The following siRNAs purchased from Dharmacon were used: ON-TARGETplus Non-targeting Pool (D-001810-10), siGENOME Human BAD siRNA - SMARTpool (M-003870-02), siGENOME Human EDF1 siRNA - SMARTpool (M-009697-01), siGENOME Human GNB2 siRNA - SMARTpool (M-017241-00), siGENOME Human HRAS siRNA - SMARTpool (M-004142-00), siGENOME Human DGUOK siRNA - SMARTpool (M-006719-03), siGENOME Human RPS6KA4 siRNA - SMARTpool (M-004664-01), siGENOME Human MAP3K12 siRNA - SMARTpool (M-003312-02), and siGENOME Human MIB2 siRNA - SMARTpool (M-015287-02). Each siRNA was used at a final concentration of 10 nM, and total siRNA amount per well was normalized across all conditions by adding non-targeting siRNA accordingly. 4 days after transfection, cell viability was assessed using the CellTiter-Glo Luminescent Cell Viability Assay (Promega, G7570) following the manufacturer’s instructions. Luminescence was measured with a SpectraMax iD3 microplate reader (Molecular Devices).

**Quantitative Reverse-transcription PCR**

MDA-MB-231 cells were seeded at a concentration of 25,000 cells/well in a 12-well format and reverse transfected with the indicated siRNAs as described above. 2 days after transfection, RNA was collected using the RNEasy Mini kit (Qiagen, 74106) and isolated according to the manufacturer’s instructions. RNA was reverse transcribed to cDNA using the iScript cDNA synthesis kit (BioRad, 1708891), and RT-qPCR was performed using Fast SYBR Green Master Mix (Applied Biosystems, 4385617) on the ViiA7 Real-Time PCR System (Applied Biosystems). Statistical tests were run in GraphPad Prism 9.2.0. All primers used for this study are listed in S3 Table.
